# Two-dimensional ultrathin Ti_3_C_2_ MXene nanosheets coated intraocular lens for synergistic photothermal and NIR-controllable rapamycin releasing therapy against posterior capsule opacification

**DOI:** 10.3389/fbioe.2022.989099

**Published:** 2022-08-30

**Authors:** Zi Ye, Yang Huang, Jinglan Li, Tianju Ma, Lixiong Gao, Huihui Hu, Qing He, Haiying Jin, Zhaohui Li

**Affiliations:** ^1^ Senior Department of Ophthalmology, The Third Medical Center, The Chinese PLA General Hospital, Beijing, China; ^2^ Department of Ophthalmology, Shanghai Electric Power Hospital, Shanghai, China; ^3^ Department of Ophthalmology, Shanghai East Hospital, Tongji University School of Medicine, Shanghai, China; ^4^ Suzhou Beike Nano Technology Co., Ltd., Suzhou, China

**Keywords:** posterior capsule opacification, rapamycin, Ti3C2 nanosheets, photothermal therapy, NIR-controlled, intraocular lens

## Abstract

Posterior capsule opacification (PCO) is one of the most frequent late-onset complications after cataract surgery. Several kinds of drug-eluting intraocular lenses (IOL) were designed for sustainable drug release to suppress ocular inflammation, the proliferation of lens epithelial cells (LECs) and the development of PCO after cataract surgery. Despite previous advances in this field, the drug-loaded IOLs were limited in ocular toxicity, insufficient drug-loading capacity, and short release time. To prevent PCO and to address these drawbacks, a novel drug-loaded IOL (Rapa@Ti_3_C_2_-IOL), prepared from two-dimensional ultrathin Ti_3_C_2_ MXene nanosheets and rapamycin (Rapa), was fabricated with a two-step spin coating method in this study. Rapa@Ti_3_C_2_ was prepared via electrostatic self-assembly of Ti_3_C_2_ and Rapa, with a loading capacity of Rapa at 92%. Ti_3_C_2_ was used as a drug delivery reservoir of Rapa. Rapa@Ti_3_C_2_-IOL was designed to have the synergistic photothermal and near infrared (NIR)-controllable drug release property. As a result, Rapa@Ti_3_C_2_-IOL exhibited the advantages of simple preparation, high light transmittance, excellent photothermal conversion capacity, and NIR-controllable drug release behavior. The Rapa@Ti_3_C_2_ coating effectively eliminated the LECs around Rapa@Ti_3_C_2_-IOL under a mild 808-nm NIR laser irradiation (1.0 W/cm^−2^). Moreover, NIR-controllable Rapa release inhibited the migration of LECs and suppressed the inflammatory response after photothermal therapy *in vitro.* Then, Rapa@Ti_3_C_2_-IOL was implanted into chinchilla rabbit eyes, and the effectiveness and biocompatibility to prevent PCO were evaluated for 4 weeks. The Rapa@Ti_3_C_2_-IOL implant exhibited excellent PCO prevention ability with the assistance of NIR irradiation and no obvious pathological damage was observed in surrounding healthy tissues. In summary, the present study offers a promising strategy for preventing PCO *via* ultrathin Ti_3_C_2_ MXene nanosheet-based IOLs with synergistic photothermal and NIR-controllable Rapa release properties.

## Introduction

Posterior capsule opacification (PCO) is a common complication and the primary cause of non-refractive visual loss after lens extraction combined with intraocular lens (IOL) implantation (Schartmuller et al., 2020; Wormstone et al., 2021). It is characterized by the proliferation, migration and epithelial-mesenchymal transformation (EMT) of residual lens epithelial cells (LECs), which eventually gives rise to matrix deposition and contraction on the posterior capsule and opacification of the visual axis. The incidence of PCO is reported to be 20%–60% in adults and 95% or more in children and adolescents (Ursell et al., 2018; Cooksley et al., 2021). Importantly, with the increasing application of multifunctional IOL, such as multifocal IOL and astigmatism corrected IOL, people have higher expectations for visual quality, and PCO increases postoperative complaints related to visual quality (Alio et al., 2017). Neodymium-doped yttrium aluminum garnet (Nd: YAG) laser has been regarded as the most effective therapeutic strategy for PCO till now (Maedel et al., 2021), while a number of potential risks are inevitable, such as IOL dislocation, mild anterior uveitis, macular edema and retinal detachment (Burq and Taqui, 2008). In addition, it is often inappropriate for children who are difficult to cooperate in the treatment.

Several strategies, including surgical techniques, IOL materials, IOL with square edge design and drug-eluting IOL, were performed for the prophylaxis of PCO (Awasthi et al., 2009; Topete et al., 2021; Liu et al., 2022a; Lu et al., 2022). The introduction of the hydrophobic IOLs with a square edge design can reduce PCO incidence, but the efficacy is limited (Leydolt et al., 2020; Maedel et al., 2021; Xiang et al., 2021; Darian-Smith et al., 2022). Recently, drug-loaded IOLs which contribute to suppressing the proliferation and migration of LECs have been an attractive strategy to prevent PCO (Zhang and Xie, 2020). For instance, researchers have designed the drug-eluting IOL with sustained bromfenac release (Zhang X. et al., 2022), anti-proliferative drug-loaded polysaccharide-modified IOL (Han et al., 2019), polylactide-glycolic acid and rapamycin (Rapa) coating IOL (Liu et al., 2009) and so on. Rapa, as a third-generation novel immunosuppressant, has the properties of anti-proliferation, anti-inflammation, low toxicity and high cell membrane penetration (Xu et al., 2022). Moreover, the effectiveness and biocompatibility of Rapa-eluting stents have been confirmed in some clinical trials (Abhyankar et al., 2018; Nathani et al., 2020; Pamidimukkala et al., 2020). Based on these data, the Rapa-laden IOL for PCO therapy has been designed and fabricated (Liu et al., 2009). Nevertheless, the drug-loaded IOLs were limited in ocular toxicity, insufficient drug-loading capacity, and short release time (Mylona and Tsinopoulos, 2020).

Several bioactive nanoparticles are applied for killing cells with photothermal effect under 808 nm near infrared (NIR) irradiation, such as graphene oxide for infected wounds (Federico et al., 2022), gold nanoparticles for mice bearing tumor (Xing et al., 2018), black phosphorus for breast cancer (Li et al., 2022), and carboxylated CuInS/ZnS quantum dots for PCO (Mao et al., 2021). Thus, killing the residual LECs with the photothermal nanomaterial integrated IOLs has become a promising candidate for PCO therapy. A novel nanoplatform-based IOL, whose rim is coated with Au@SiO_2_ nanorods, was fabricated for eliminating residual LECs after cataract surgery (Lin Y. X. et al., 2017). After NIR irradiation (3.0 W/cm^2^), the PCO occurrence is reduced to 30–40% in a rabbit model. However, as nanorods were coated on the rim of IOLs, the temperature in the center of IOL gradually decreased to 38°C, which would not reach the temperature of killing the proliferating LECs in the optics of IOLs. Therefore, it is essential to find a material with high light transmittance to evenly coat the whole optic surface of IOL for the photothermal ablative effect on the optic axis. Moreover, photothermal therapy (PTT) gives rise to the inflammatory response (Xu et al., 2018), which is an important cause of PCO (Stepp and Menko, 2021). This may also be one of the reasons for the limitation of PTT alone in PCO treatment. Therefore, the NIR light-controllable drug release system, using nano-carrier materials with a high photothermal conversion effect to control drug release under the action of NIR, would be an attractive approach. In addition, the animal models of PCO used in many studies choose New Zealand rabbits, whose retinal pigment epithelial (RPE) cells and iris lack pigment (Lin Y. X. et al., 2017; Tang et al., 2021). The NIR power in these studies may not be safe to be applied in human eyes. Thus, it is essential to establish a chinchilla rabbit model of PCO.

Recently, MXene, the two-dimensional transition metal carbides/carbonitrides, has been wildly applied in biomedical fields, owing to the unique ultrathin planar nanostructure, and corresponding physiochemical properties (Lu et al., 2021; VahidMohammadi et al., 2021; Alwarappan et al., 2022; Szuplewska et al., 2022; Wang et al., 2022). MXene nanosheets, such as Ti_3_C_2_, are featured with high photothermal-conversion capacity, which is developed as an effective photothermal nanoplatform upon NIR irradiation. Moreover, the ultrathin planar nanostructure of MXene provides a large anchoring site for numerous drug molecules. Therefore, in the field of biomedicine, MXenes make themselves outstanding in biosensors, antibacterial activity, drug delivery, photothermal therapy and hemodialysis (Rasool et al., 2016; Lin et al., 2017a). Interestingly, ultrathin Ti_3_C_2_T_x_ has an optic property of high light transmittance, which is superior to that of carbon nanotubes and graphene (Zhang et al., 2017; VahidMohammadi et al., 2021). It has been coated on the surface of commercial IOL to form an accommodating IOL with an easy two-step spin coating method without affecting the optical performance or quality of IOL (Ward et al., 2020). Furthermore, MXene is suitable as the photothermal transducer for NIR-controllable drug release, which is attributed to the localized surface plasma resonance effect (Beckers et al., 2021; Dong et al., 2021). However, the biocompatibility of MXenes remains controversial, which inhibits the further application of MXenes in biomedicine. The biological properties of MXenes relate to their carbon and/or nitrogen content, which are the main building blocks of all living organisms (Rozmyslowska-Wojciechowska et al., 2020). Whereas, early transition metals such as Ti, Ta and Nb, are stated as relatively inert, however, increasing reports suggest their potential toxicity (Jastrzebska et al., 2017). Due to these properties, MXene-coated IOL may be an attractive strategy for PCO prevention by synergistic photothermal effect and NIR-controllable drug release behavior, while the biosafety should be carefully evaluated both *in vitro* and *in vivo*.

In the present study, we designed and fabricated novel ultrathin Ti_3_C_2_ MXene nanosheets coated IOL loading with Rapa (Rapa@Ti_3_C_2_-IOL), namely Rapa@Ti_3_C_2_-IOL, for PCO prevention. The ultrathin Ti_3_C_2_ nanosheets were prepared by wet etching the Ti_3_AlC_2_ MAX phase. Then, Rapa-loaded Ti_3_C_2_ nanosheets (Rapa@Ti_3_C_2_) were synthesized *via* electrostatic self-assembly and were coated onto the optic surface of the commercial hydrophobic IOL by an easy two-step spin coating method. This Rapa@Ti_3_C_2_-IOL showed the performance of simple preparation, high light transmittance, excellent photothermal ablative efficacy, and NIR-controllable Rapa release behavior, which effectively prevents PCO both *in vitro* and *in vivo*. This study presented the therapeutic effect of Rapa@Ti_3_C_2_-IOL upon NIR irradiation and provided a promising application for PCO prevention.

## Materials and methods

### Materials

Ti_3_AlC_2_ (powder, 400-meshes) was bought from Beijing Forsman Scientific (China). Ethanol absolute (≥99.7%) and HF (40%, purity >40.0%) were bought from Sinopharm Chemical Reagent Co., Ltd. (Shanghai, China). Tetrapropylammonium hydroxide (TPAOH) (25 wt.% in water) and Rapa were bought from Sinopharm Chemical Reagent Co., Ltd. All chemicals were used as received without further treatment unless otherwise stated.

### Synthesis of Ti_3_C_2_

According to the methods reported previously (Lin et al., 2017a; Gou et al., 2019). Briefly, 2 g of Ti_3_AlC_2_ powder was etched at 10 ml 40% HF at room temperature to remove the Al layer. The precipitate was then centrifuged three times in turn with water and ethanol for 3 min at 3500 rpm. The monolayer Ti_3_C_2_ nanosheets were intercalated with 20 ml TPAOH(25 wt. % in water)and were vacuum-dried and stored at 4°C.

### Preparation of Rapa@Ti_3_C_2_

Monolayer Ti_3_C_2_ was dispersed in phosphate buffered saline (PBS = 7.2) to prepare suspensions of different concentrations. A certain amount of Rapa was added to the Ti_3_C_2_ dispersion and stirred for 2 h. Then, the Rapa-loaded Ti_3_C_2_ nanosheets (Rapa@Ti_3_C_2_) were collected by centrifugation for 3 min at 3500 rpm and stored at 4°C. The absorption peak intensity of Rapa was measured by UV-vis–NIR spectrometer, and the absorption peak was observed at 278 nm. The loading capacity of Rapa can be calculated according to:

$$\mathrm{Loading}\;\mathrm{capacity}\left(\%\right)=\frac{\mathrm{Weight}\;\mathrm{of}\;\mathrm{Rapa}\;\mathrm{in}\;\mathrm{Rapa@T}i_{3}C_{2}}{\mathrm{Weight}\;\mathrm{of}\;Ti_{3}C_{2}}\times 100\%$$ (1)

The Rapa loading capacity was 92%.

### Fabrication of Ti_3_C_2_-IOL and Rapa@Ti_3_C_2_-IOL

As described previously (Ward et al., 2020), the Ti_3_C_2_ solution and Rapa@Ti_3_C_2_ solution (the concentration ratio of Rapa/Ti_3_C_2_ = 0.92:1) were coated onto the optic surface of hydrophobic acrylic IOLs supplied by Eyebright Medical Technology (Beijing) Co., Ltd. *via* a two-step spin coating method. In brief, the loops of IOLs were shielded with scotch tape and the optic surface of hydrophobic IOLs was cleaned and activated with oxygen plasma by an OKSUN plasma etcher (OKSUN Technology Co., Ltd., Guangzhou, China). 40 μL of Ti_3_C_2_ or Rapa@Ti_3_C_2_ solution was evenly deposited on the optic surface by a spin coater (SanYan Technology Co., Ltd., Shanghai, China) at 5,000 rpm for 2 min, followed by drying the coating at 6000 rpm for 1 min. There are three types of IOLs in this study: C-IOLs = commercial IOLs as the control, Ti_3_C_2_-IOLs = IOLs were coated with Ti_3_C_2_; Rapa@Ti_3_C_2_-IOLs = IOLs were coated with Rapa@Ti_3_C_2_.

### Characterizations

Ti_3_C_2_ MXenes and Rapa@Ti_3_C_2_ were characterized by Scanning Electron Microscopy (SEM, Sigma300), Transmission Electron Microscope (TEM, FEI Tecnai G2 F20) and Atomic Force Microscope (AFM, Bruker Dimension Icon). The formation of Ti_3_C_2_ was also confirmed by X-ray diffraction (XRD, Panalytical X'Pert'3 Powder). Size and zeta potential were acquired by Malvern Zetasizer Nano ZS90 (Britain). UV–vis absorption spectra were captured by a SHIMADZU UV3600 spectrometer.

### Cumulative drug release assay

When evaluating NIR-controlled drug release, the Rapa@Ti_3_C_2_ solution was irradiated by a NIR laser (808 nm). The absorbance of the solution was determined at λ = 278 nm at given time intervals to calculate the Rapa releasing percentage.

### Photothermal effect and photostability *in vitro*

Rapa@Ti_3_C_2_ solution at different concentrations was directly irradiated by NIR light at different powers density for 180 s. The temperatures of Rapa@Ti_3_C_2_ solution (25 μg/ml) with different laser power densities ranging from 0.1 to 3.0 W/cm^2^ were recorded by an infrared thermal imager (UNI-T, UTi120S). The temperatures of Rapa@Ti_3_C_2_-IOLs with various concentrations of Ti_3_C_2_ (0, 5, 10, 25, 50 μg/ml) were detected under irradiation with different power densities. Finally, the photothermal stability of Rapa@Ti_3_C_2_-IOL was evaluated by three cycles of the laser on/off. The changes in temperature were recorded every 10 s by an infrared thermal imager.

### Cytotoxicity and proliferation assay of lens epithelial cells

The anterior capsule of the C57 mouse (8 weeks, 20–25 g) was cut into a square of 1 mm × 1 mm and cultured in Dulbecco’s modified Eagle’s medium (DMEM) containing 2% fetal bovine serum. Once the cells grew out of the capsule, they were digested and harvested for subculture. NIH/3T3 (CRL-1658) was obtained from American Type Culture Collection (ATCC), and cultured in the same condition.

The CCK-8 assay kit (Dojindo) was used for testing the cytotoxicity of Rapa@Ti_3_C_2_. Mouse LECs and NIH/3T3 cells were seeded in the 96-well plate at 8 × 10^3^ per well in 2% FBS-supplemented and 1% penicillin/streptomycin-supplemented DMEM. After being cultured for 24 h, a fresh medium containing free Rapa, free Ti_3_C2, or Rapa@Ti_3_C_2_ at different concentrations (0, 5, 10, 25, 50, 100, 500 μg/ml) was added with or without the 808 nm laser irradiation. Then, the CCK-8 solution was diluted 1:10 with DMEM for evaluating the cytotoxicity.

### Calcein-AM/PI double staining assay

To evaluate the photothermal ablative effect of nano-IOLs, three types of IOLs were carefully put on top of the cells seeded in 24 well plates and irradiated by NIR laser (1.0 W/cm^2^) for 10 min. After that, calcein-AM/PI double-staining of live/dead assay was used to label cells for 10 min and washed three times with PBS. Finally, cells were observed and captured by a fluorescence microscope.

### Wound healing assay

About 1 × 10^6^ mouse LECs were seeded in 24-well plates and cultured with IOL for 24 h. They were divided into 5 groups as follows: 1) C-IOL; 2) Ti_3_C_2_-IOL; 3) Ti_3_C_2_-IOL + NIR; 4) Rapa@Ti_3_C_2_-IOL; 5) Rapa@Ti_3_C_2_-IOL + NIR. After the adhesion of cells, a sterile pipette tip was used to scratch the adhesion surface to evaluate cell migration, and the images were captured by microscopy at 0 h, 24 h and 48 h after scratching. ImageJ software was used to measure the wound area: more than three random fields were selected for evaluation, and the wound area at 0 h time point was taken as the reference.

### Transwell assay

The Transwell assay was performed *via* Transwell migration chambers (8 μm pore size) from BD Biosciences. Cells were grown to 70% confluence and serum was starved for 24 h. Following detachment with trypsin and washing with PBS, cells were re-suspended in serum-free medium and added to the upper chamber. Serum-free medium containing various solutions or IOLs were added to the bottom chamber. After 24 and 48 h, the cells that had not migrated were removed from the upper face of the filters with cotton swabs, and the migrated cells were fixed and stained with crystal violet/methanol solution.

### Cytokines analysis *in vitro*

In our previous study, the inflammatory cytokines (IL-1β, IL-6, TNF-α, and MMP-9) were determined by using the Multiplex Luminex Assay (Milliplex Multiplex Assays Luminex xMAP, Merck, Darmstadt, Germany) with Multiplex kits (Millipore). In brief, 1 × 10^6^ mouse LECs were seeded in 24-well plates and cultured with C-IOL, Ti_3_C_2_-IOL and Rapa@Ti_3_C_2_-IOL upon NIR irradiation (1.0 W/cm^2^) for 10 min. After 24 h, the supernatant was collected for analysis. 50 μL of the sample was incubated with the primary antibodies against IL-1β, IL-6, TNF-α, and MMP-9 at 4°C. The standard curve ranges from 3.2 to 10,000 pg/ml.

### Animal model of posterior capsule opacification

All animal experiments were performed following the guidelines of the ARVO Statement for the Use of Animals in Ophthalmic and Vision Research (NIH publication No. 85-23, revised 1985) and approved by the Laboratory Animal Ethics Committee of the Third Medical Center of PLA General Hospital. Sixty female chinchilla rabbits up to 2.0–2.5 kg weight, 12 weeks old were used during the experiment, which was randomly divided into five groups (*n* = 12 rabbits/group): 1) C-IOL; 2) Ti_3_C_2_-IOL; 3) Ti_3_C_2_-IOL + NIR; 4) Rapa@Ti_3_C_2_-IOL; 5) Rapa@Ti_3_C_2_-IOL + NIR. All animals were anesthetized by intravenous injection of 3% pentobarbital sodium at 1 ml/kg body weight, and preoperative pupil dilation was performed with 1 drop of 0.5% tropicamide (Santen, Japan) 3 times, 15 min apart. Following that, they all underwent phacoemulsification and IOL implantation in the left eyes by the same surgeon (Y, Huang). A cornea incision was established by keratome. Phacoemulsification was performed with the Stellaris system (Bausch & Lomb, NY), and different groups of IOL were implanted respectively.

Postoperative ophthalmic medications included levofloxacin eye drops (Santen, Japan) and dexamethasone-tobramycin (Alcon Laboratories, Inc., Belgium) four times daily for 2 weeks. After surgery, a slit lamp photography system (Topcon slit lamp and camera, Topcon Corp. Tokyo, Japan) was used to observe and photograph the experimental eyes at designated time points. The degrees of PCO was assessed using photographs by retro illumination according to the criteria listed in Supplementary Table S1 (Vargas et al., 2003). The inflammatory response in the anterior segment is assessed by the severity of the aqueous flare according to the criteria listed in Supplementary Table S2 (Vargas et al., 2002).

### Photothermal effect *in vivo*

One week after surgery, the eyes of rabbits were irradiated by NIR laser (808 nm, 1.0 W/cm^2^) for 10 min. The changes in temperature of implanted IOL were recorded every 10 s by a thermal imager.

### Histology

At the end of the experiment, the rabbits were sacrificed. The degree of PCO was observed and captured from the posterior side of the eye (Miyake-Apple view). Furthermore, a histological examination of different ocular tissues was carried out, and the posterior capsules, corneas, ciliary bodies and retinas were evaluated using hematoxylin and eosin (H&E) staining.

### Evaluation of rapa release in aqueous humor

To detect the release of Rapa, 0.1 ml aqueous humor was collected in a centrifuge tube at day 1, 3, 8, 15, 22, and 29 after surgery. The concentration of Rapa in the aqueous humor was determined by high performance liquid chromatography (HPLC, Gilson) at a wavelength of 278 nm.

### Intraocular pressure, central corneal thickness, and corneal endothelial cells measurement

A TonoVet Plus^®^ tonometer (Icare Finland Oy, Helsinki, Finland) was used to measure intraocular pressure (IOP). Anterior segment optical coherence tomography was used to measure the central corneal thickness (CCT). Corneal specular microscopy was used to count the number of corneal endothelial cells (CEC) (SP·3000P, Topcon, Japan). All experiments were repeated three times and the average value was recorded.

### Statistical analysis

The data were analyzed using Graphpad Prism software version 9.2 and expressed as the mean ± standard error of mean. One-way ANOVA with post-hoc testing was used for comparison of multiple groups with Gaussian distribution. Kruskal–Wallis test was used for comparison of multiple groups without normal distribution. *p* < 0.05 was considered statistically significant.

## Results and discussion

### Preparation and characterization of Rapa@Ti_3_C_2_

**SCHEME 1:**
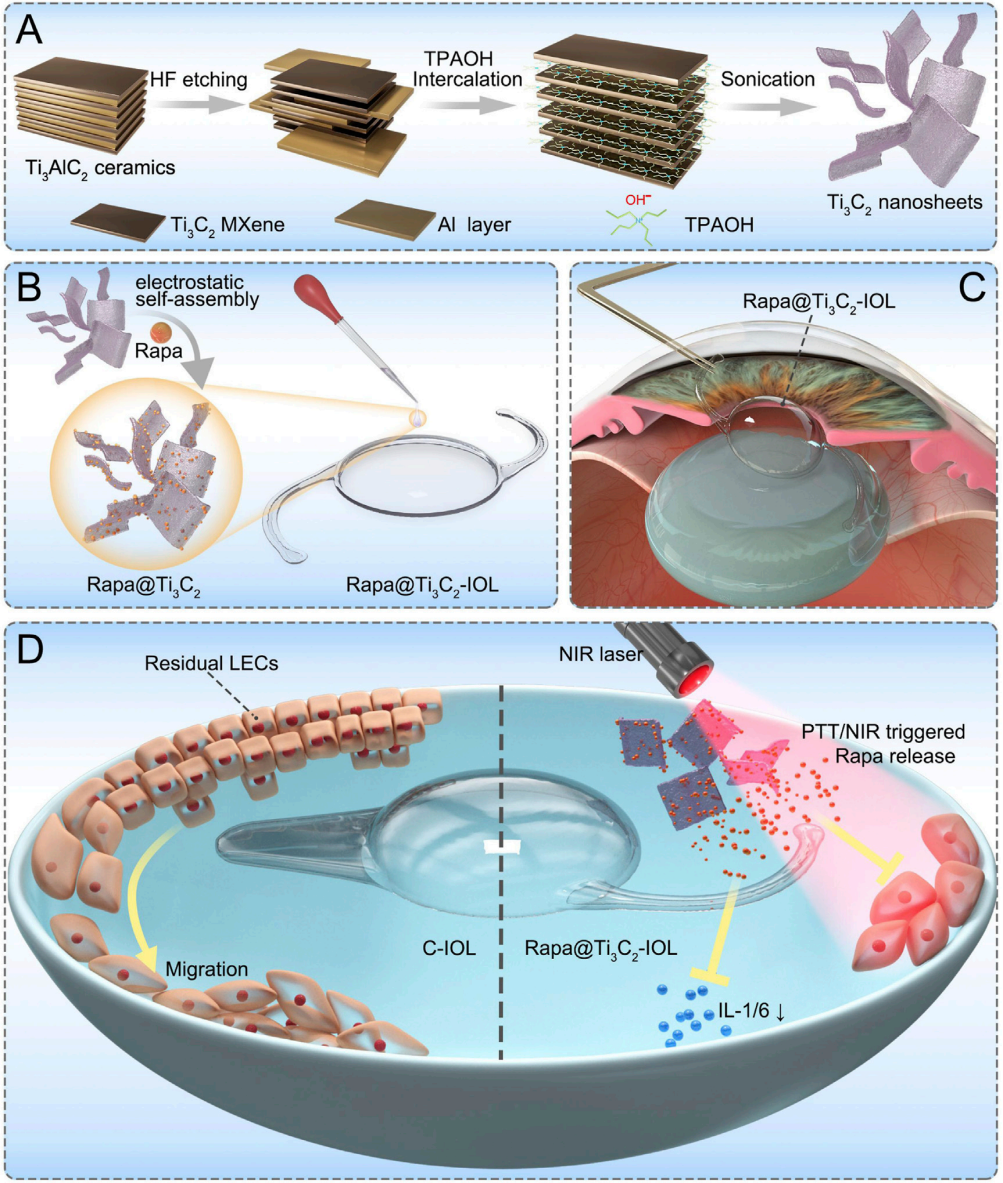
The scheme of Rapa@Ti_3_C_2_-IOL for synergistic photothermal and NIR-controllable Rapa releasing therapy for preventing PCO. **(A)** The synthetic process of Ti_3_C_2_ nanosheets. **(B)** The fabrication of Rapa@Ti_3_C_2_-IOL with a two-step spin coating method. **(C)** The implantation of Rapa@Ti_3_C_2_-IOL in a rabbit model of PCO. **(D)** The synergistic photothermal and NIR-controllable Rapa releasing effect of Rapa@Ti_3_C_2_-IOL against PCO.

Scheme 1 illustrates the working mechanism of the NIR light-controllable drug-releasing system, which enables the tunable on-demand release of drugs from Rapa@Ti_3_C_2_-IOL and photothermal conversion temperature. Ultrathin Ti_3_C_2_ MXene nanosheets were synthesized using HF etching and a TPAOH intercalator, which could produce nano-sized MXene nanosheets with high dispersion and sufficient planar size to meet the severe requirements for current and future biomedical applications (Zhou et al., 2021; Korupalli et al., 2022). Ti_3_C_2_ nanosheets were further developed as drug delivery nanosystems due to their ultrathin planar nanostructures and corresponding large surface area. Then, Rapa, as a potent immunosuppressive drug with anti-proliferation and anti-inflammation effects, was loaded on the Ti_3_C_2_ nanosheets using the electrostatic self-assembly method. Due to the negative charge on the surface of Ti_3_C_2_ nanosheets and the positive charge on Rapa molecules, a high drug loading capacity could be achieved (Huang et al., 2022; Korupalli et al., 2022; Pires et al., 2022). The Ti_3_C_2_ nanosheets and Rapa-Ti_3_C_2_ nanosheets thus prepared have abundant functional groups on their surfaces, which can be easily dispersed in PBS (Supplementary Figures S1A,B). The Ti_3_C_2_ solution with a concentration of 25 μg/ml is transparent, which is used as the Ti_3_C_2_ concentration with Ti_3_C_2_-IOL and Rapa@Ti_3_C_2_-IOL.

**FIGURE 1:**
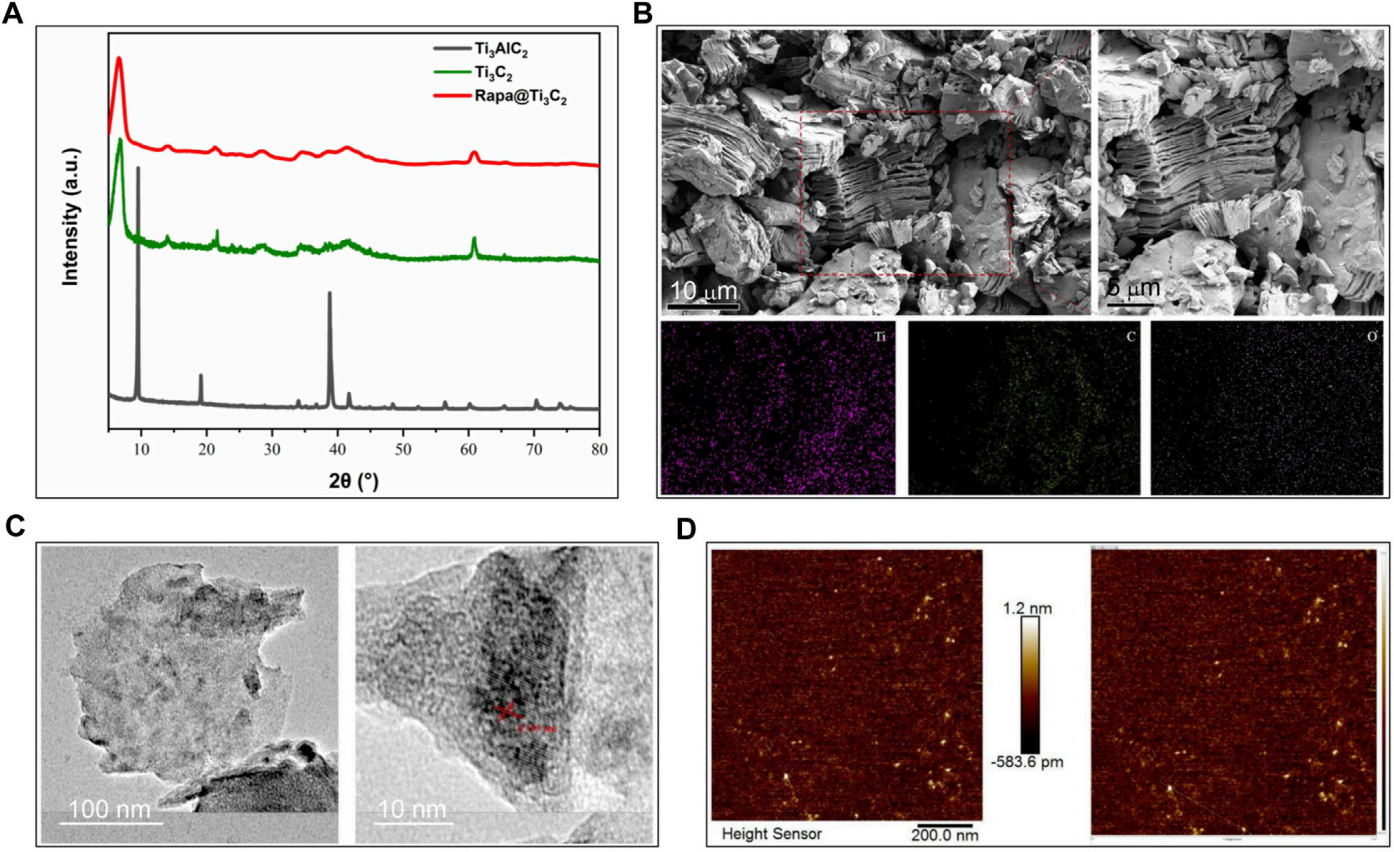
Characterization of Ti_3_C_2_ and Rapa@Ti_3_C_2_ nanosheets. **(A)** The X-ray diffraction peak of Ti_3_AlC_2_, Ti_3_C_2_ and Rapa@Ti_3_C_2_. **(B)** The layer structures stacked into bulks of Ti_3_C_2_ MXene after HF etching in SEM images. **(C)** The planar sheet morphology of highly dispersed Ti_3_C_2_ MXene in AFM images. **(D)** The crystallized structure of Ti_3_C_2_ MXene in TEM images.

The morphology of Ti_3_C_2_ nanosheets was examined by SEM, TEM and AFM. After TPAOH exfoliation, the most obvious XRD peak of Ti_3_AlC_2_ (2θ ≈ 38°) disappears and is replaced by the characteristic periodic peak originating from stacked Ti_3_C_2_ layers. Namely, Ti_3_C_2_ and Rapa@ Ti_3_C_2_ only have peak values at 2θ ≈ 6° (Figure 1A). The SEM images show the structure of multilayer Ti_3_C_2_ MXene with a typical layer after HF etching (Figure 1B). Next, after stripping by TPAOH, monolayer Ti_3_C_2_ MXene nanosheets were produced with the characteristic of high dispersion. AFM is a typical method for characterizing the planar structure of two-dimensional materials, and it demonstrates the typical two-dimensional sheet morphology of highly dispersed Ti_3_C_2_ MXene (Figure 1C). The high-resolution TEM images show that these Ti_3_C_2_ MXene nanosheets have high crystal crystallinity, which was revealed by crystalline lattice and indicates that the two-step exfoliation method did not affect the crystal structure of Ti_3_C_2_ MXene (Figure 1D).

**FIGURE 2:**
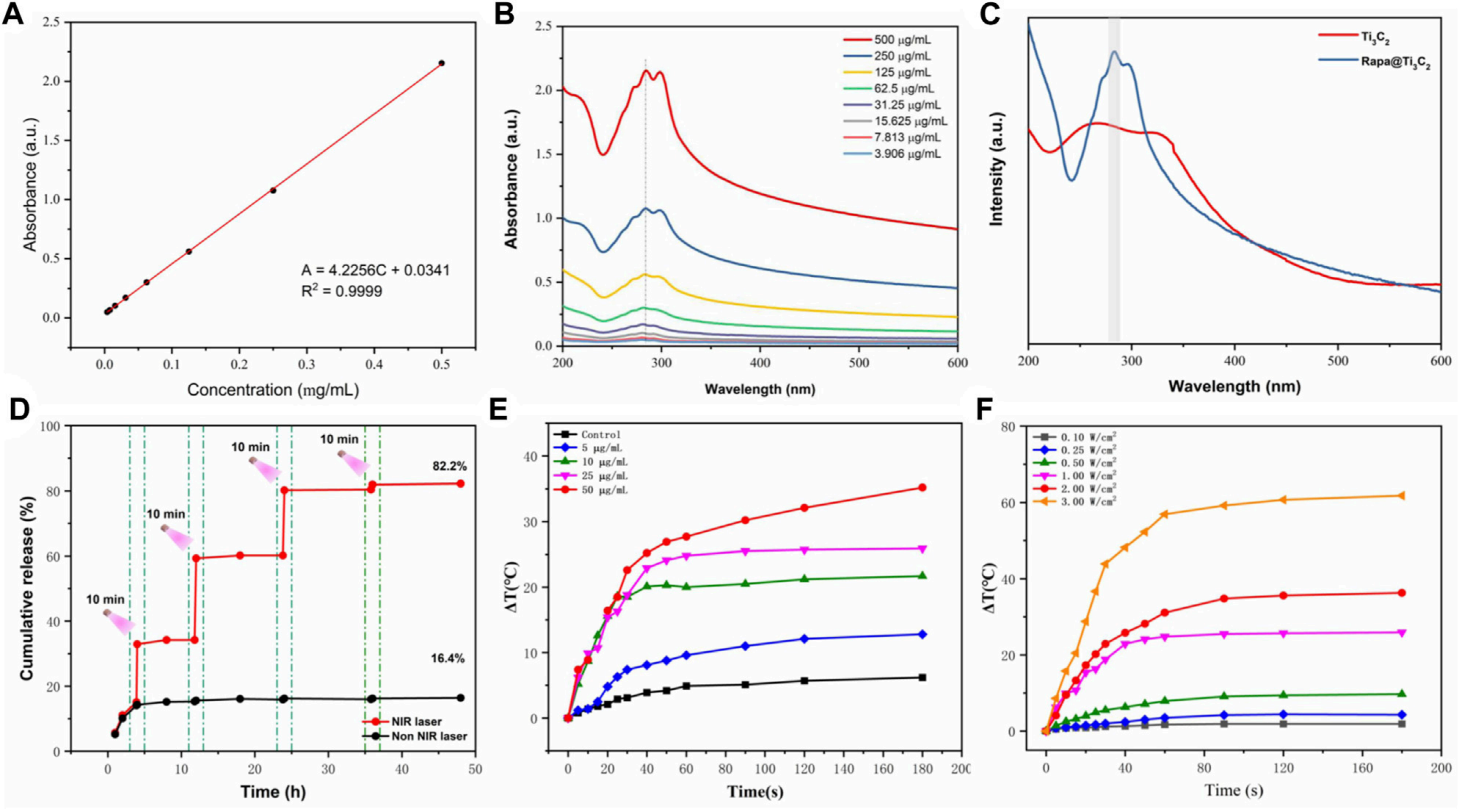
*In vitro* photothermal performance and drug release of Rapa@Ti_3_C_2_ nanosheets. **(A)** The extinction coefficient of Rapa@Ti_3_C_2_ calculated by the linear fit of concentration of Ti_3_C_2_ and A/L at 808 nm. **(B)** UV–vis spectrum of Rapa@Ti_3_C_2_ nanosheets with different concentrations of Ti_3_C_2_. **(C)** UV–vis spectrum of Ti_3_C_2_ and Rapa@Ti_3_C_2_ nanosheets, exhibiting the characteristic absorption peaks of Rapa molecules in 278 nm. **(D)** The cumulative Rapa releasing rates of Rapa@Ti_3_C_2_ nanosheets with or without NIR irradiation (1 W/cm^2^) were calculated after 48 h, respectively. **(E)** The temperatures of different concentrations of Rapa@Ti_3_C_2_ upon 808 nm NIR irradiation (1 W/cm^2^) for 180 s. **(F)** The temperatures of Rapa@Ti_3_C_2_ (25 μg/ml) upon different power densities of NIR irradiation for 180 s.

The extinction coefficient of Rapa@Ti_3_C_2_ was calculated by the linear fit based on the concentration (0, 7.813, 15.625, 31.25, 62.5, 125, 250, 500 μg/ml) of Ti_3_C_2_ and absorbance at 808 nm (Figure 2A). Following the formula of Lambert-Beer law:

$$\frac{A}{L}=\alpha C$$ (2)

(A: the absorbance of Ti_3_C_2_ at 808 nm, L: path-length = 1 cm, α: the extinction coefficient, C: the concentration of Ti_3_C_2_), the extinction coefficient of Rapa@Ti_3_C_2_ was calculated to be 4.2256 L g^−1^cm^−1^ (A = 4.2256C + 0.0341, *R* ^2^ = 0.9999), indicating its probability to be used in further PTT. UV–vis spectrum showed that Ti_3_C_2_ nanosheets loaded with Rapa exhibited characteristic absorption peaks of Rapa molecules (λ = 278 nm) (Figures 2B,C), indicating that Rapa was successfully loaded onto Ti_3_C_2_ nanosheets. The NIR irradiation could trigger Rapa release from Ti_3_C_2_ nanosheets for 3 cycles, and the cumulative Rapa-releasing rate reached 82.2% after 48 h (Figure 2D). Especially at the irradiation time point, the Rapa releasing was remarkably increased due to the local heating effect resulting from PTT mediated by Ti_3_C_2_. Therefore, Ti_3_C_2_ as a novel drug-delivery nanosystem could achieve the function of NIR-enhanced drug releasing because of its unique surface characteristic and photothermal conversion characteristic (Lin et al., 2017a; Han et al., 2018; Wang et al., 2021). The photothermal conversion power of Rapa@Ti_3_C_2_ was assessed by observing the temperature rise of Rapa@Ti_3_C_2_ solution irradiated by the 808 nm laser. The temperature increases with the increase of Ti_3_C_2_ concentrations (0, 5, 10, 25, and 50 μg/ml) and power density of irradiation (0.1, 0.25, 0.5, 1.0, 2.0, 3.0 W/cm^2^) (Figures 2E,F), indicating that Ti_3_C_2_ can be used for photothermal conversion to increase the temperature of the environment around it.

### The effects of Ti_3_C_2_ and Rapa@Ti_3_C_2_ on photothermal ablation, cell proliferation and migration *in vitro*

*In vitro* cytotoxicity of Ti_3_C_2_ and Rapa@Ti_3_C_2_ was further evaluated by CCK-8 assay (Rozmyslowska-Wojciechowska et al., 2020; Wu et al., 2020). After incubating mouse LECs and NIH/3T3 (CRL-1658) with Rapa, Ti_3_C_2_, and Rapa@Ti_3_C_2_ at different concentrations for 24 h, the results showed that Ti_3_C_2_ or Rapa@Ti_3_C_2_ at a concentration of 25 μg/ml had no obvious cytotoxic effect on both cells, verifying the biosafety of Ti_3_C_2_, which was consistent with previous studies (Rozmyslowska-Wojciechowska et al., 2020; Wu et al., 2020). However, Ti_3_C_2_ or Rapa@Ti_3_C_2_ at a concentration of 25 μg/ml led to significant cytotoxicity of LECs and NIH/3T3 upon NIR laser irradiation (1 W/cm^2^) for 10 min (Supplementary Figure S2). When LECs and NIH/3T3 were incubated with Ti_3_C_2_ or Rapa@Ti_3_C_2_ (the concentration of Ti_3_C_2_ was 25 μg/ml) upon NIR irradiation at different power densities for 10 min, nearly no cell alive at the power density ≥1.0 W/cm^2^ (Supplementary Figure S3).

Rapa, a potent immunosuppressive drug, can inhibit the proliferation and migration of LECs *in vitro* (Liu et al., 2009). After incubation of LECs and NIH/3T3 with free Rapa or Rapa@Ti_3_C_2_ (the ratio of Rapa/Ti_3_C_2_ = 0.92:1) at different concentrations of Rapa for 72 h, The number of cells decreased significantly when the free Rapa concentration ≥1.0 μg/ml, while the anti-proliferative effect of Rapa@Ti_3_C_2_ is inferior to that of free Rapa in the same concentration (Supplementary Figure S4). Therefore, the concentration of Ti_3_C_2_ with 25 μg/ml and laser power density of 1.0 W/cm^2^ was chosen for the next step of investigation.

The impact of Rapa and Rapa@Ti_3_C_2_ on the migration ability of mouse LECs and NIH/3T3 cells was evaluated *via* Transwell assay. It was obvious that the number of migrated cells in Rapa and Rapa@Ti_3_C_2_ groups were all significantly lower than that in the control group (Supplementary Figure S5). Moreover, the number of migrated cells and migration rate was lower in the free Rapa group than that in the Rapa@Ti_3_C_2_ group, suggesting that Rapa plays a major role in inhibiting migration, and Ti_3_C_2_ restricts the release of Rapa. All these results demonstrated that the Rapa@Ti_3_C_2_ may be a potential nanosystem with a synergistic photothermal effect and drug release behavior in preventing PCO.

### Characterization of Ti_3_C_2_-IOL and Rapa@Ti_3_C_2_-IOL

**FIGURE 3:**
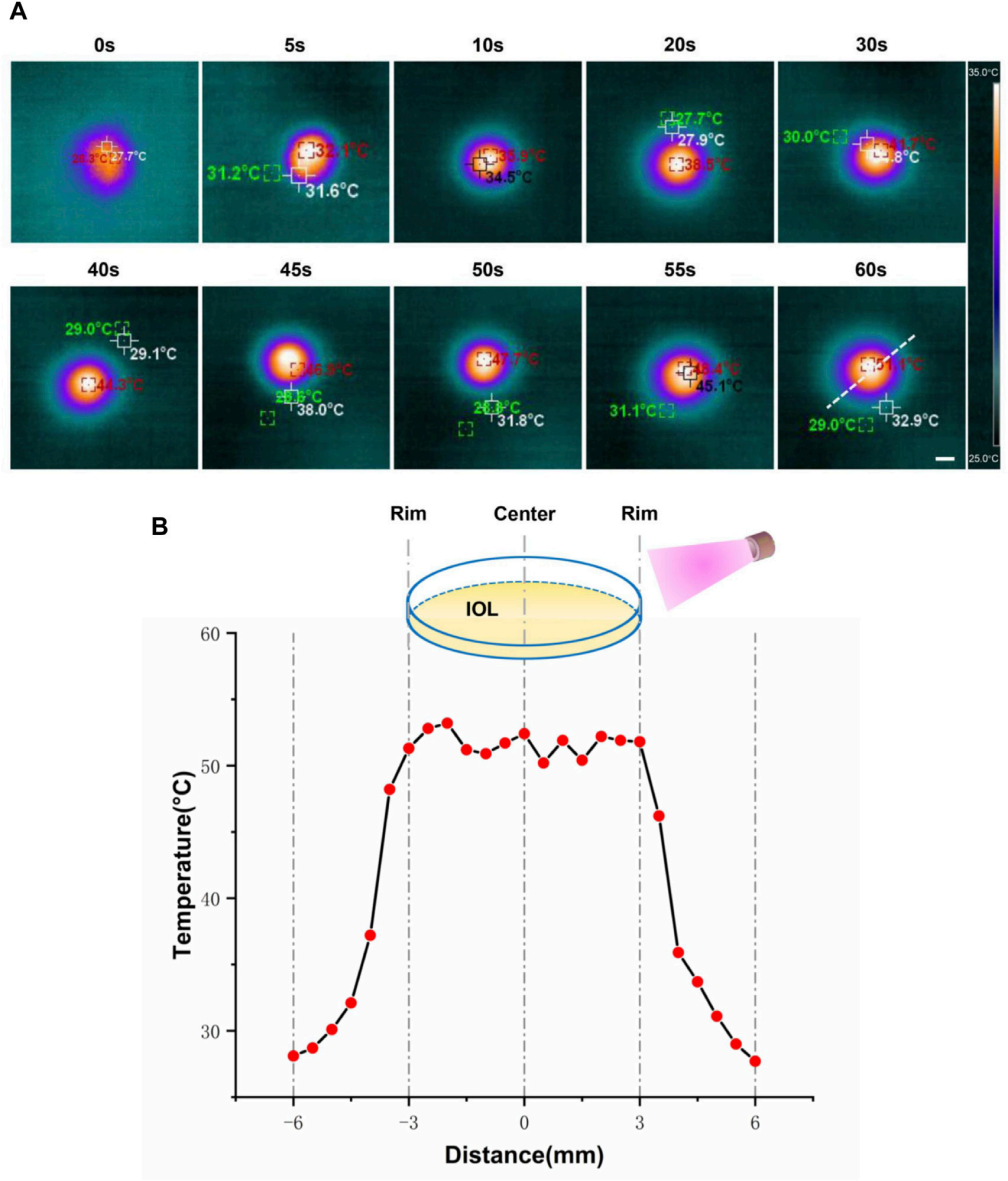
*In vitro* photothermal performance of Rapa@Ti3C2-IOL. **(A)** Rapa@Ti_3_C_2_-IOLs were irradiated by 808 nm NIR (1 W/cm^2^) for 60 s, and the temperatures of Rapa@Ti_3_C_2_-IOLs were recorded by infrared thermal imager every 5 s. **(B)** The temperature of the optical region of IOL with a diameter of 6 mm could reach more than 50°C, and the temperature of the region outside the optical region decreased to below 30°C.

The photothermal effect of Rapa@Ti_3_C_2_-IOL upon NIR irradiation was studied by a thermal imager. The Rapa@Ti_3_C_2_-IOLs were irradiated by 808 nm laser and the thermal images were captured every 5 s. The optical region of Rapa@Ti_3_C_2_-IOLs changed gradually from orange to yellow, indicating the increase of temperature under irradiation over time (Figure 3A). The temperature of the optical region of IOL with a diameter of 6 mm could reach more than 50°C, while the temperature of the region outside the optical region rapidly decreased to below 30°C (Figure 3B), indicating that the photothermal conversion capacity of Ti_3_C_2_ was comparable to the other biomaterials (Ying-Yan et al., 2021; Liu et al., 2022b) and the photothermal effect of Rapa@Ti_3_C_2_-IOL is effective. As described in the previous studies, the distance between the cornea and the posterior capsule is about 7 mm, and approximately 18 mm from the posterior capsule to the retina, which ensures the safety of Rapa@Ti_3_C_2_-IOL without damage to surrounding ocular tissues (Sorenson et al., 2000; Toni et al., 2010).

**FIGURE 4:**
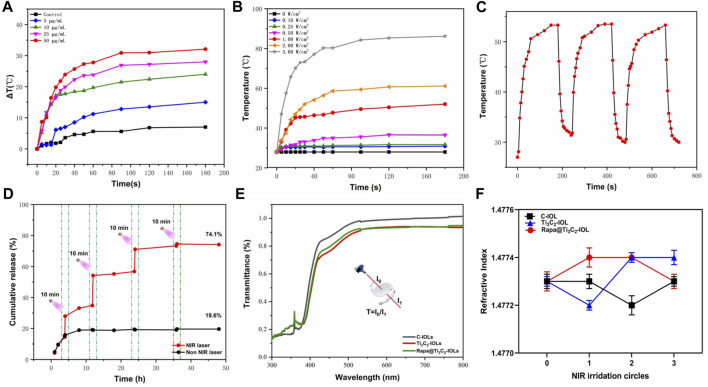
*In vitro* photothermal performance, drug release, light transmittance and refractive power of Rapa@Ti_3_C_2_-IOL. **(A)** The temperatures of different concentrations of Rapa@Ti_3_C_2_-IOL upon 808 nm NIR irradiation (1 W/cm^2^) for 180 s. **(B)** The temperatures of Rapa@Ti_3_C_2_-IOL (25 μg/ml) upon different power density of NIR irradiation (1 W/cm^2^) for 180 s. **(C)** Heating curves of Rapa@Ti_3_C_2_-IOL for three cycles of laser on/off (1.0 W/cm^2^) upon NIR irradiation. The temperature enhancement kept almost the same for each heating process. **(D)** The cumulative Rapa releasing rates of Rapa@Ti_3_C_2_-IOL with or without NIR irradiation (1 W/cm^2^) were calculated after 48 h, respectively. **(E)** The light transmittance of the optical regions of C-IOL, Ti_3_C_2_-IOL and Rapa@Ti_3_C_2_-IOL. **(F)** The refractive index of C-IOL, Ti_3_C_2_-IOL and Rapa@Ti_3_C_2_-IOL for three cycles of laser on/off (1.0 W/cm^2^) upon NIR irradiation.

To detect photothermal capacity, temperatures of Rapa@Ti_3_C_2_-IOL under different power densities of 808 nm laser irradiation were recorded by the thermal imager. That data revealed that the temperature increased with the elevation of Ti_3_C_2_ concentrations and the power density of irradiation (Supplementary Figure S6), and the temperature of Rapa@Ti_3_C_2_-IOL was enhanced significantly and reached a plateau after NIR irradiation for 90 s (Figures 4A,B). The temperature of Rapa@Ti_3_C_2_-IOL increased from 28°C to above 50°C. Similarly, the heating stability of Rapa@Ti_3_C_2_-IOL was further studied by three cycles of laser on/off (1.0 W/cm^2^) upon NIR irradiation. The temperature range of each heating process kept the same, indicating that Rapa@Ti_3_C_2_-IOL has high thermal stability (Figure 4C). Meanwhile, the cumulative Rapa releasing rate from Rapa@Ti_3_C_2_-IOL was detected. The cumulative Rapa release rate from Rapa@Ti_3_C_2_-IOL was 19.6% without NIR exposure at 48 h. In contrast, the NIR laser could trigger Rapa release, and the Rapa-releasing rate reached 74.1% after 4 times of NIR irradiation at 48 h (Figure 4D). The increased release rate of Rapa during NIR irradiation was due to the photothermal effect mediated by Ti_3_C_2_. Therefore, Rapa@Ti_3_C_2_-IOL could achieve the combinational photothermal effect and NIR-controllable drug release behavior.

As an implantable optical material, the IOLs should be analyzed in the optical properties and surface morphology (Tang et al., 2021). Supplementary Figure S7 showed the images of the IOL before and after being coated with different concentrations of Rapa@Ti_3_C_2_, indicating that the optical region of Rapa@Ti_3_C_2_-IOLs was transparent when Ti_3_C_2_ concentration was below 50 μg/mL. As shown in Figure 4E, the light transmittance of the pristine material was nearly 90% in the optical region when the concentration of Ti_3_C_2_ was 25 μg/ml. The refractive index of IOL is another critical factor that affects the imaging properties (Park and Jang, 2017). There was no significant difference in refractive index among C-IOL, Ti_3_C_2_-IOL and Rapa@Ti_3_C_2_-IOL after three cycles of laser on/off (1.0 W/cm^2^) upon NIR irradiation (Figure 4F). Furthermore, the breaking elongation of Rapa@Ti_3_C_2_-IOL was 0.98 MPa, and the elastic modulus was 95.21%, which was nearly the same as that of C-IOLs and Ti_3_C_2_-IOL (Supplementary Figure S8). These experimental results indicated that the ultrathin Rapa@Ti3C2 coated IOLs had high light transmittance and excellent mechanical properties.

### Photothermal ablative and anti-inflammatory effect of Rapa@Ti_3_C_2_-IOL *in vitro*

**FIGURE 5:**
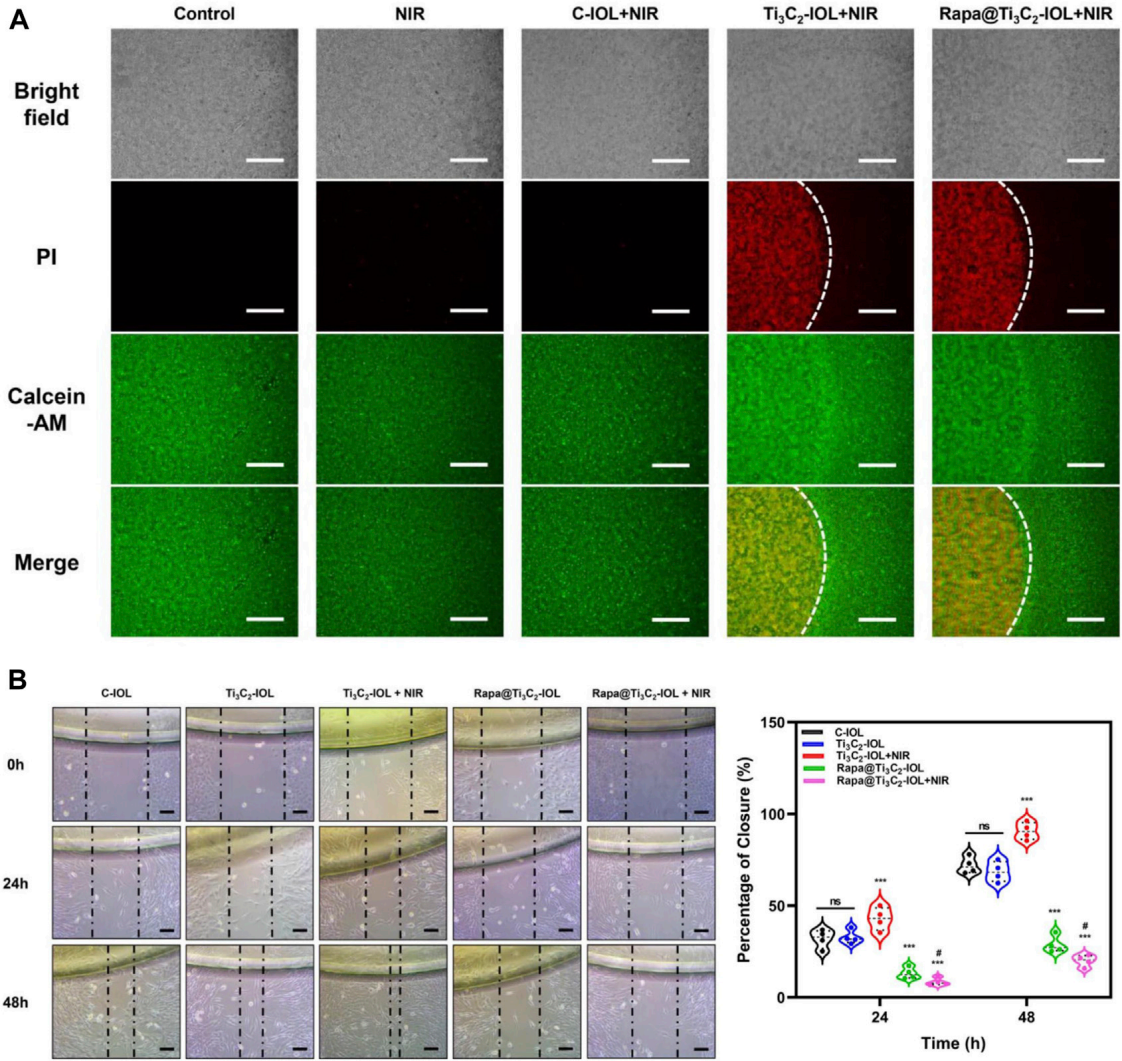
Photothermal ablative and anti-migrative effect of Rapa@Ti_3_C_2_-IOL *in vitro*. **(A)** Live/dead dual staining of LECs. The cells covered by Ti_3_C_2_-IOL and Rapa@Ti_3_C_2_-IOL irradiated with NIR laser (1 W/cm^2^) for 10 min. **(B)** The wound healing assay showed the effects of different IOLs on LECs migration. Scale bar = 500 μm.

Several previous studies confirmed that 50°C for 8–10 min upon NIR irradiation was effective for the region-selective killing of nano-IOLs (Lin Y. X. et al., 2017; Mao et al., 2021; Zhang C. et al., 2022). To evaluate the photothermal ablative effect of nano-IOLs in the present study, three types of IOLs were gently placed on the top of LECs in a 24-well plate. After 10 min irradiation of NIR laser (1.0 W/cm^2^), the cells were tested for dual live/dead staining assay. Fluorescence microscope images showed that the cells covered by Ti_3_C_2_-IOL or Rapa@Ti_3_C_2_-IOL were dead and stained with red signals, while cells away from the IOL were alive and stained with green signals (Figure 5A). It was obvious that about 99% of cells in the Ti_3_C_2_-IOL or Rapa@Ti_3_C_2_-IOL covered region were dead. In contrast, in the control groups including the Control, NIR, and C-IOL + NIR group, all the cells were alive. These data indicated that the region-confined killing effect of Ti_3_C_2_-IOL and Rapa@Ti_3_C_2_-IOL on LECs was due to the photothermal effect.

Several studies have reported that Rapa prevents PCO progression by inhibiting inflammatory response, cell proliferation, migration, extracellular matrix formation, and EMT (Liu et al., 2009; Liu et al., 2010; Meng et al., 2013). To investigate the anti-migrative effects of Rapa released from Rapa@Ti_3_C_2_-IOL, the cells were treated with 5 different conditions as follows: 1) C-IOL; 2) Ti_3_C_2_-IOL; 3) Ti_3_C_2_-IOL + NIR; 4) Rapa@Ti_3_C_2_-IOL; 5) Rapa@Ti_3_C_2_-IOL + NIR. At the time point of 0 h, 24 h and 48 h after scratch, the changes in cell morphology and scratch wound areas were observed. The wound-healing assay displayed that the area of the remaining scratch wounds was much wider in the Rapa@Ti_3_C_2_-IOL + NIR group than that of the non-Rapa group, indicating that Rapa significantly suppressed cell migration in mouse LECs (Figure 5B). Moreover, the anti-migrative effect of Rapa on LECs was further tested by Transwell assay. The data suggested that the Rapa@Ti_3_C_2_-IOL + NIR group had the least number of migrated cells (Supplementary Figure S9). Several inflammatory cytokines, such as IL-1β, IL-6, TNF-α, and MMP-9 are associated with PCO development (Konopinska et al., 2021). We found that the levels of IL-1β, IL-6 and TNF-α were significantly higher in the cell culture medium of the Ti_3_C_2_-IOL + NIR group compared with the control group, as PTT gives rise to the inflammatory response. However, after NIR irradiation of Rapa@Ti_3_C_2_-IOL, the levels of IL-1β, IL-6 and TNF-α reduced to the baseline, which confirms the NIR-controllable Rapa release behavior (Supplementary Figure S10). All these results suggested that the Rapa@Ti_3_C_2_-IOL has the synergistic photothermal ablative effect and NIR-controllable Rapa release behavior *in vitro*.

### Rapa@Ti_3_C_2_-IOL for PCO prevention *in vivo*

**FIGURE 6:**
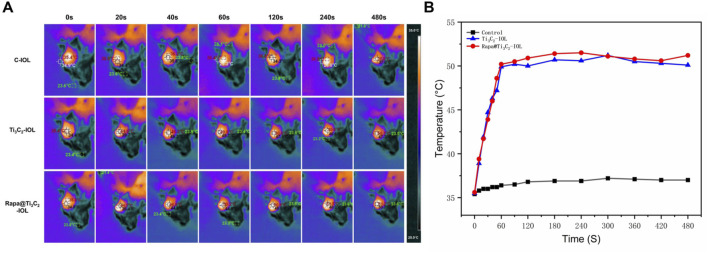
*In vivo* photothermal performance of Rapa@Ti_3_C_2_-IOL. **(A)** IOLs in different groups were implanted into chinchilla rabbits’ left eyes, and the temperatures were recorded by thermal imager. **(B)** The temperature of Ti_3_C_2_-IOL and Rapa@Ti_3_C_2_-IOL increased with the irradiation time and rapidly rose to the peak temperature at 1 min.

**FIGURE 7:**
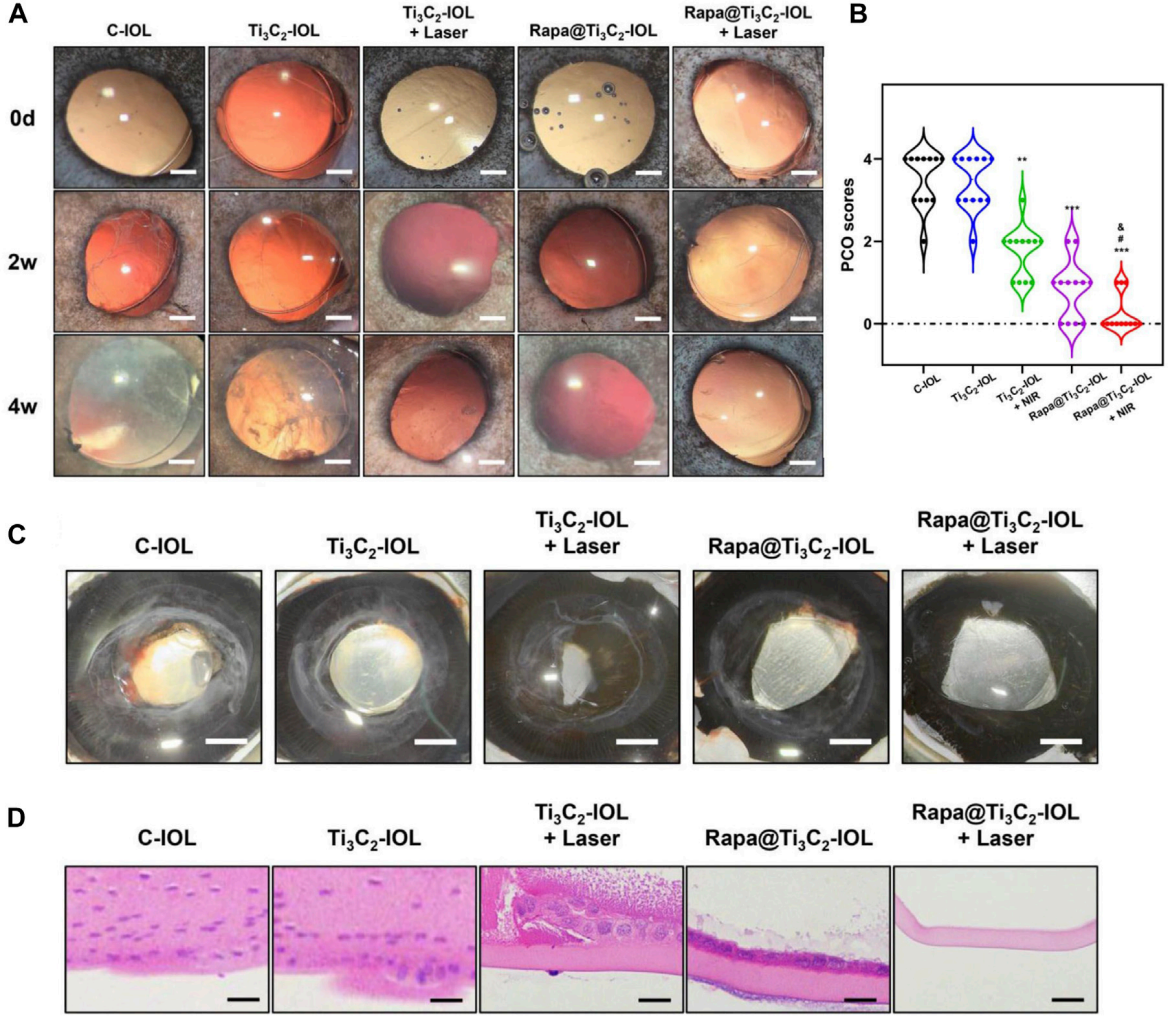
Rapa@Ti_3_C_2_-IOL implantation for PCO prevention *in vivo*. **(A)** The slit-lamp images of anterior segments of rabbits underwent IOL implantation on day 14 and day 28 after lens extraction surgery (Scale bar = 1 mm). **(B)** The PCO scores in different groups on day 28. **(C)** The Miyake-Apple view of the eyes after surgery on day 28 (Scale bar = 1 mm). **(D)** H&E staining of posterior capsules in different groups (Scale bar = 50 μm).

To evaluate the effect of Rapa@Ti_3_C_2_-IOL on PCO prevention *in vivo*, the chinchilla rabbit model of PCO was established and evaluated according to previous reports (Zhang et al., 2016; Lin et al., 2017b). All rabbits underwent phacoemulsification and IOL implantation in the left eyes. The NIR laser (808 nm, 1.0 W/cm^2^, 10 min) was irradiated once a week post operation, and the temperature of IOL was recorded by a thermal imager in the first week after surgery. The data showed that the temperature of Ti_3_C_2_-IOL and Rapa@Ti_3_C_2_-IOL rapidly rose to 50°C in the 1st min after NIR irradiation (Figure 6). The slit-lamp microscope was used to observe anterior segments of rabbit eyes at 0, 2, and 4 weeks after the operation. In the C-IOLs and Ti_3_C_2_-IOL groups, it was obvious that residual LECs grow and migrate to the visual axis on the posterior capsules from the 2nd week after surgery, while the posterior capsules of the Rapa@Ti_3_C_2_-IOL + NIR group remained clear. In the Ti_3_C_2_-IOL + NIR and Rapa@Ti_3_C_2_-IOL groups, the proliferative LECs were less on the posterior capsules compared to those in the C-IOLs and Ti_3_C_2_-IOL groups. Nevertheless, the proliferative tissues were still more significant than those in the Rapa@Ti_3_C_2_-IOL + NIR group (Figure 7A). In the Rapa@Ti_3_C_2_-IOL + NIR group, all rabbits had a PCO score no more than grade 1, while at least 5 of 12 rabbits had a PCO score as grade 2 or above in the other groups (Figure 7B). The mean grade of PCO in the Rapa@Ti_3_C_2_-IOL + NIR group was (0.25 ± 0.13), remarkably better than that of the C-IOL group (3.50 ± 0.19), Ti_3_C_2_-IOL group (3.42 ± 0.19), Ti_3_C_2_-IOL + NIR group (1.75 ± 0.17) and Rapa@Ti_3_C_2_-IOL group (0.83 ± 0.21). On day 28 post-operation, the Miyake-Apple view of the rabbits’ eyes showed no obvious proliferation materials on the posterior capsules of the Rapa@Ti_3_C_2_-IOL + NIR group (Figure 7C). On the 28th day after surgery, the eyeballs of the rabbits were harvested for histological assay. There were obvious proliferative tissues on the posterior capsules in the C-IOL group, Ti_3_C_2_-IOL group, Ti_3_C_2_-IOL + NIR group and Rapa@Ti_3_C_2_-IOL group (Figure 7D). Nevertheless, few cells were on the posterior capsules in the Rapa@Ti_3_C_2_-IOL + NIR group.

The Rapa concentrations in aqueous humor after Rapa@Ti_3_C_2_-IOL implantation were tested by HPLC. The Rapa concentration in the Rapa@Ti_3_C_2_-IOL group was (1.19 ± 0.08) µg/ml on day 1 after surgery and decreased gradually. From the 15th day, Rapa was not detectable in aqueous samples. However, 1 day after NIR irradiation, the Rapa concentration in aqueous humor increased to about 3 μg/ml on day 8, day 15 and day 22. As NIR irradiation triggered Rapa release from Ti_3_C_2_ nanosheets for 3 cycles, the Rapa concentration in aqueous humor was nearly 1 μg/ml on day 29 (Supplementary Figure S11). A high level of Rapa in aqueous humor can effectively suppress ocular inflammation and PCO progression. On day 29, the mean grade of flare in the Rapa@Ti_3_C_2_-IOL + NIR group was (0.08 ± 0.29), remarkably better than that of the C-IOL group (0.75 ± 0.62), Ti_3_C_2_-IOL group (0.67 ± 0.49), Ti_3_C_2_-IOL + NIR group (1.67 ± 0.65) and Rapa@Ti_3_C_2_-IOL group (0.42 ± 0.52) (Supplementary Figure S12). All these results suggested that synergistic PTT and NIR-controllable Rapa releasing therapy effectively kills residue LECs, inhibits cell proliferation and migration, ameliorates inflammatory response in the anterior chamber and prevents PCO progression after cataract surgery.

We further assessed the histological changes of the retina under NIR irradiation with different power densities. The H&E staining of the retina in each group showed no significant changes with mild NIR power density (≤1.0 W/cm^2^), compared with that of the control group. However, the subretinal fluid (local neuroepithelial detachment) was observed in groups exposed to the laser power at 2 and 3 W/cm^2^ (Supplementary Figure S13), indicating that the NIR power density ≤1.0 W/cm^2^ was safe for ocular treatment, and this NIR power density was much lower than that in several previous studies (Lin Y. X. et al., 2017; Zhang C. et al., 2022; Liu et al., 2022b). Moreover, the morphology of cornea, iris and retina in the Rapa@Ti_3_C_2_-IOL + NIR group was not significantly different from those of the control group, C-IOL group, Ti_3_C_2_-IOL group, Ti_3_C_2_-IOL + NIR group and Rapa@Ti_3_C_2_-IOL group at day 28 after surgery (Supplementary Figure S14). In addition, we detected the changes in IOP, central corneal thickness and corneal endothelial cell density after surgery. The IOP levels were mildly elevated in the Rapa@Ti_3_C_2_-IOL group and Rapa@Ti_3_C_2_-IOL + NIR group than those of the control group on day 3 and dropped to the baseline from day 7. However, the IOP levels in the C-IOL group, Ti_3_C_2_-IOL group and Ti_3_C_2_-IOL + NIR group were significantly higher compared to those of the control group on day 28 after surgery (Supplementary Figure S15A). In contrast, the central corneal thickness and corneal endothelial cell density in the Rapa@Ti_3_C_2_-IOL + NIR group were the same as those of the control group on day 28 after surgery (Supplementary Figures S15B,C). The results confirmed the high efficiency, excellent biocompatibility and safety of the Rapa@Ti_3_C_2_-IOL, which may be an attractive strategy for future clinical applications for preventing PCO.

## Conclusion

In this study, we coated Rapa@Ti_3_C_2_ onto the surface of the optical part of commercial IOL, namely Rapa@Ti_3_C_2_-IOL, for combinational PTT and NIR-controllable drug releasing therapy for PCO prevention. This Rapa@Ti_3_C_2_-IOL system showed the characteristics of high light transmittance, moderate refractive index and comparable strain stress and elongation performances to commercial hydrophobic IOL. Notably, the Rapa@Ti_3_C_2_-IOL underwent controllable Rapa release behavior under 808 nm NIR irradiation, due to the high photothermal conversion efficiency of Ti_3_C_2_. The NIR-triggered Rapa release was beneficial for inhibiting the migration of LECs and suppressing the inflammatory response after photothermal therapy. The synergistic therapy studies of Rapa@Ti_3_C_2_-IOL show that BP- DOX@IOL possesses an excellent LECs killing ability and PCO prophylaxis both *in vitro* and *in vivo*. In addition, Rapa@Ti_3_C_2_-IOL had negligible toxicity for other intraocular tissues, which makes it promising for clinical translation. In summary, the present study provides evidence that this Rapa@Ti_3_C_2_-IOL nanosystem with excellent therapy efficiency and high biocompatibility may be applied as a promising strategy for preventing PCO.

## Data Availability

The original contributions presented in the study are included in the article/Supplementary Material, further inquiries can be directed to the corresponding authors.
