# Supplementary material for: Two-dimensional ultrathin Ti3C2 MXene nanosheets coated intraocular lens for synergistic photothermal and NIR-controllable rapamycin releasing therapy against posterior capsule opacification
Source: Front Bioeng Biotechnol. 2022 Aug 30;10:989099. doi: 10.3389/fbioe.2022.989099 (PMC9468448; doi:10.3389/fbioe.2022.989099)
Supplement: Supplementary file 1 [file DataSheet1.docx]

***Supplementary Material***

**Two-dimensional ultrathin Ti_3_C_2_ MXene nanosheets coated intraocular lens for synergistic photothermal and NIR-controllable rapamycin releasing therapy against posterior capsule opacification**

Zi Ye^1†^, Yang Huang^2,3†^, Jinglan Li^1†^, Tianju Ma^1^, Lixiong Gao^1^, Huihui Hu^4****^, Qing He^4***^, Haiying Jin^3**^ and Zhaohui Li^1*^

1 Senior Department of Ophthalmology, The Third Medical Center, The Chinese PLA General Hospital, Beijing, China

2 Department of Ophthalmology, Shanghai Electric Power Hospital, Shanghai, China

3 Department of Ophthalmology, Shanghai East Hospital, Tongji University School of Medicine, Shanghai, China

4 Suzhou Beike Nano Technology Co. Ltd., Suzhou, Jiangsu province, China.

^†^ Zi Ye, Yang Huang and Jinglan Li contributed equally to this work.

* Corresponding author: Zhaohui Li, Senior Department of Ophthalmology, The Third Medical Center, The Chinese PLA General Hospital, 69 Yongding Road, Haidian District, Beijing, China; [doctorlzhyk301@163.com](mailto:doctorlzhyk301@163.com)

** Haiying Jin, Department of Ophthalmology, Shanghai East Hospital, Tongji University School of Medicine, 150 Jimo Road, Pudong New Area, Shanghai, China; [jinhaiying666@163.com](mailto:jinhaiying666@163.com)

*** Qing He, Suzhou Beike Nano Technology Co. Ltd., 8 Dongwang Road, Suzhou, Jiangsu province, China; 2608169765qq.com

********Huihui Hu, Suzhou Beike Nano Technology Co. Ltd., 8 Dongwang Road, Suzhou, Jiangsu province, China; [2695306848@qq.com](mailto:2695306848@qq.com)

**Supplementary Table 1: The degree of PCO**

| Grade | Characteristics |
| --- | --- |
| 0 | none, without PCO |
| 1 | slight, PCO exists, while does not reach the edge of the optic |
| 2 | moderate, PCO reaches the edge |
| 3 | pronounced, PCO beyond the edge, while the visual axis is transparent |
| 4 | severe, PCO covers the visual axis |

**Supplementary Table 2: The degree of aqueous flare**

| Grade | Characteristics |
| --- | --- |
| 0 | without aqueous flare |
| 1 | slight |
| 2 | medium, with iris and crystalline lens, identified |
| 3 | obvious, with iris and crystalline lens difficult to identify |
| 4 | severe, with aqueous humor coagulated and massive cellulose exudation |


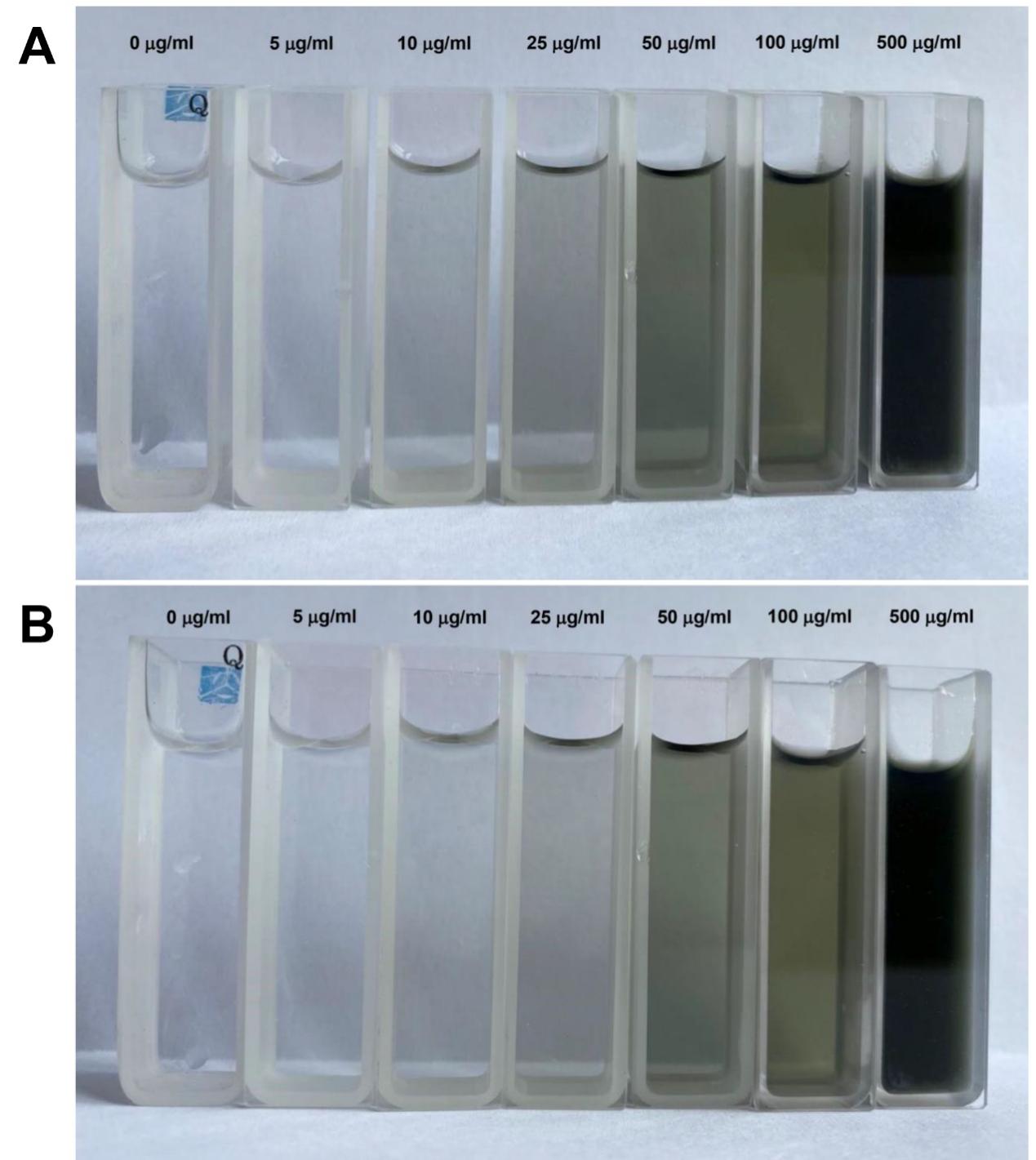


**Supplementary Figure 1.** Different concentrations of Ti_3_C_2_ (A) and Rapa@Ti_3_C_2_ (B) were dispersed in PBS.


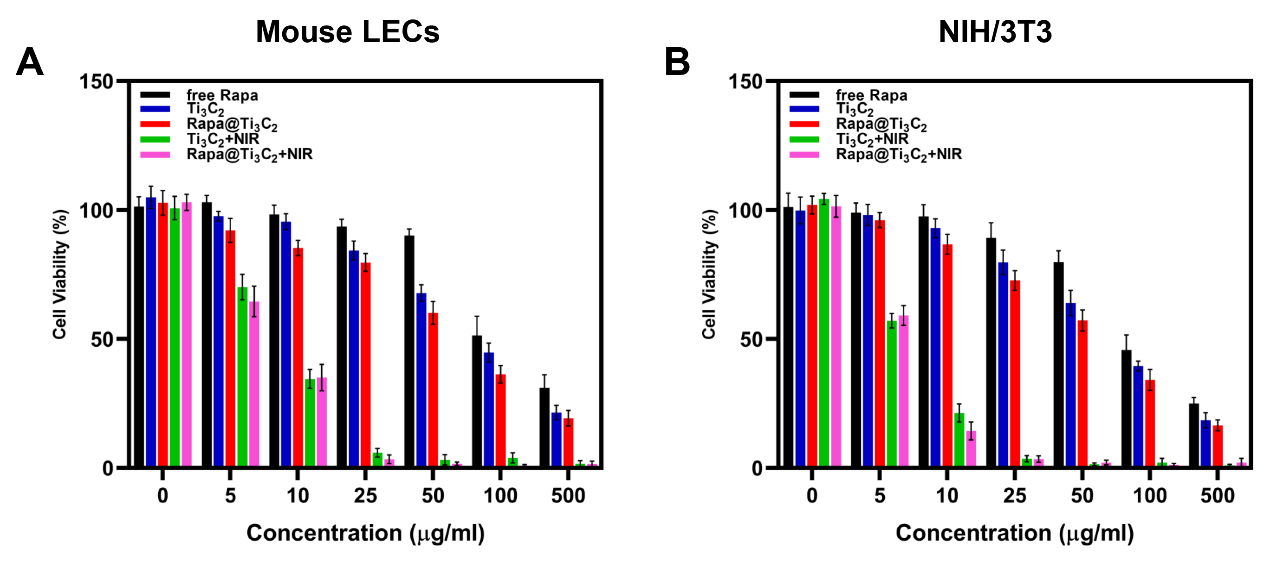


**Supplementary Figure 2**. Cytotoxic effect of Ti_3_C_2_ and Rapa@Ti_3_C_2_ at different concentrations with 1.0 W/cm^2^ NIR power on mouse LECs (A) and NIH/3T3 cells (B). N = 5.


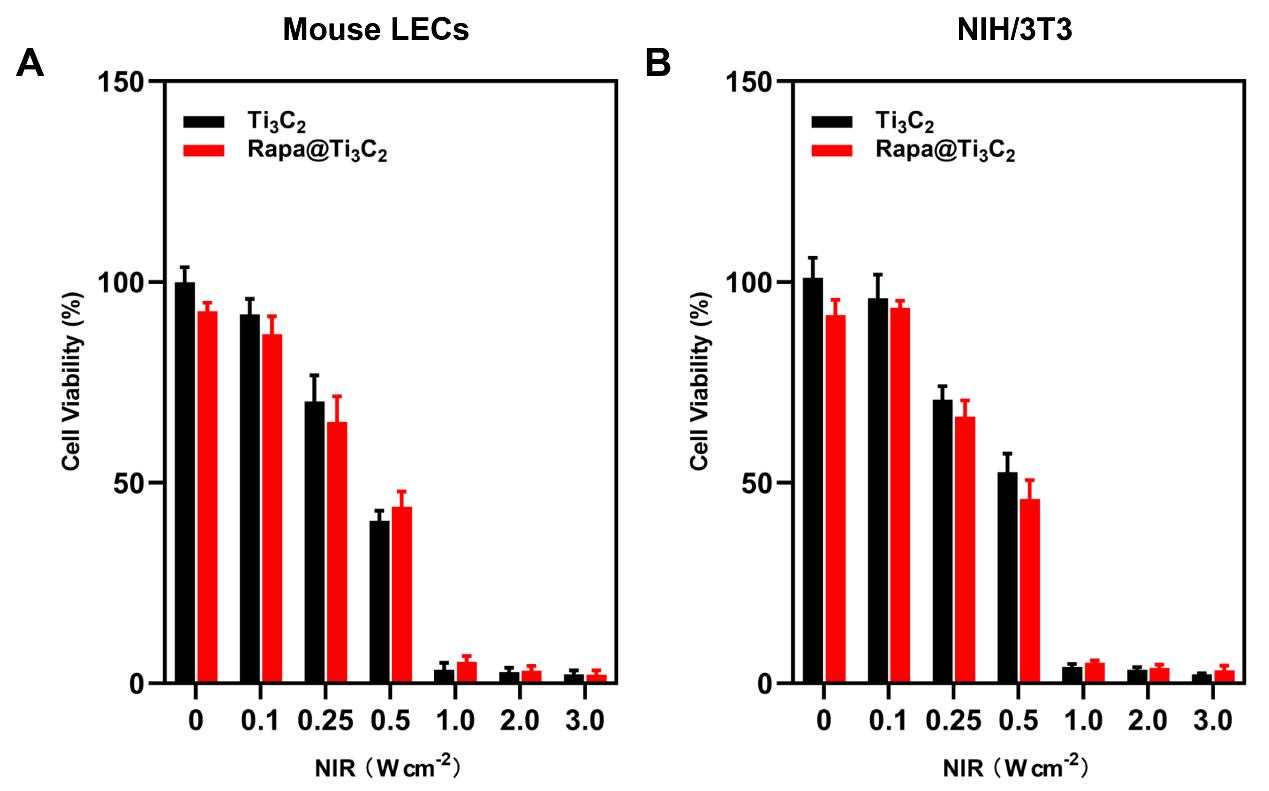


**Supplementary Figure 3**. Cytotoxic effect of Ti_3_C_2_ and Rapa@Ti_3_C_2_ with various NIR power densities on mouse LECs (A) and NIH/3T3 cells (B). N = 5.


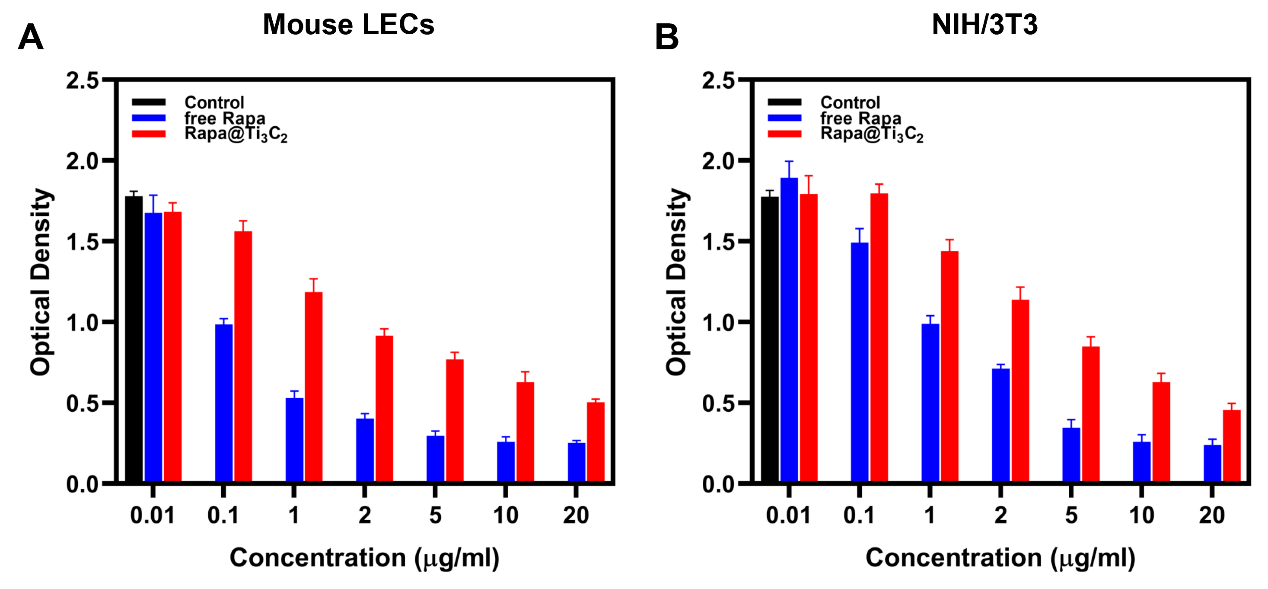


**Supplementary Figure 4**. Cytotoxic effects of Rapa and Rapa@Ti_3_C_2_ at different concentrations on mouse LECs (A) and NIH/3T3 cells (B). N = 5.


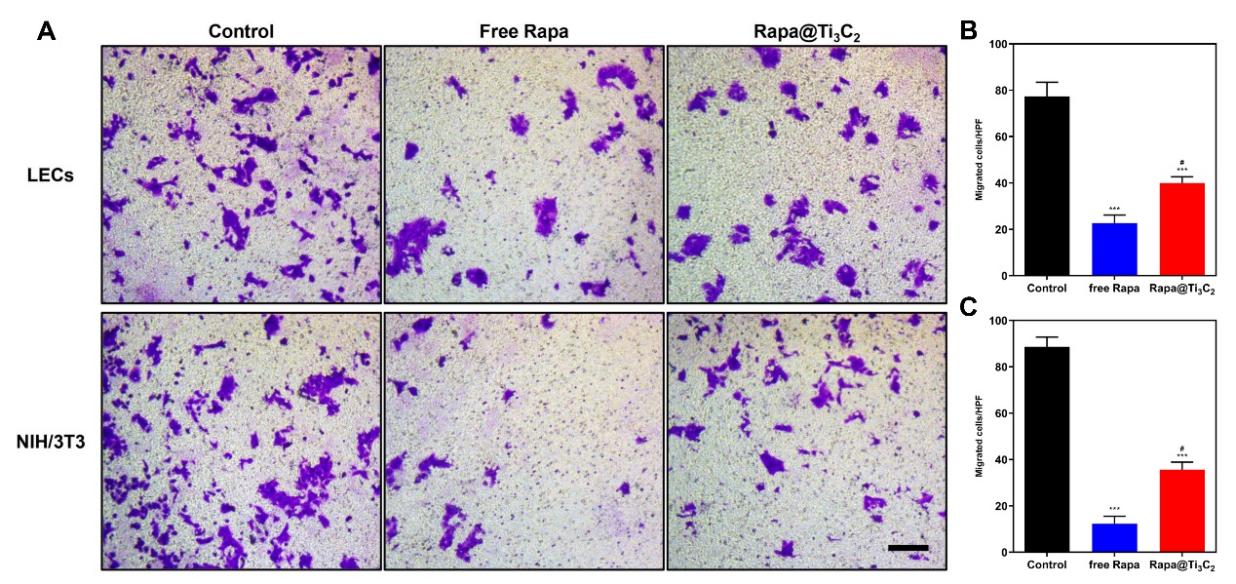


**Supplementary Figure 5**. The impact of free Rapa or Rapa@Ti_3_C_2_ on the migration ability of mouse LECs and NIH/3T3 cells using Transwell migration assay. *** p<0.001 versus the control group. # p<0.001 versus free Rapa group. N = 5. Scale bar = 50 µm,


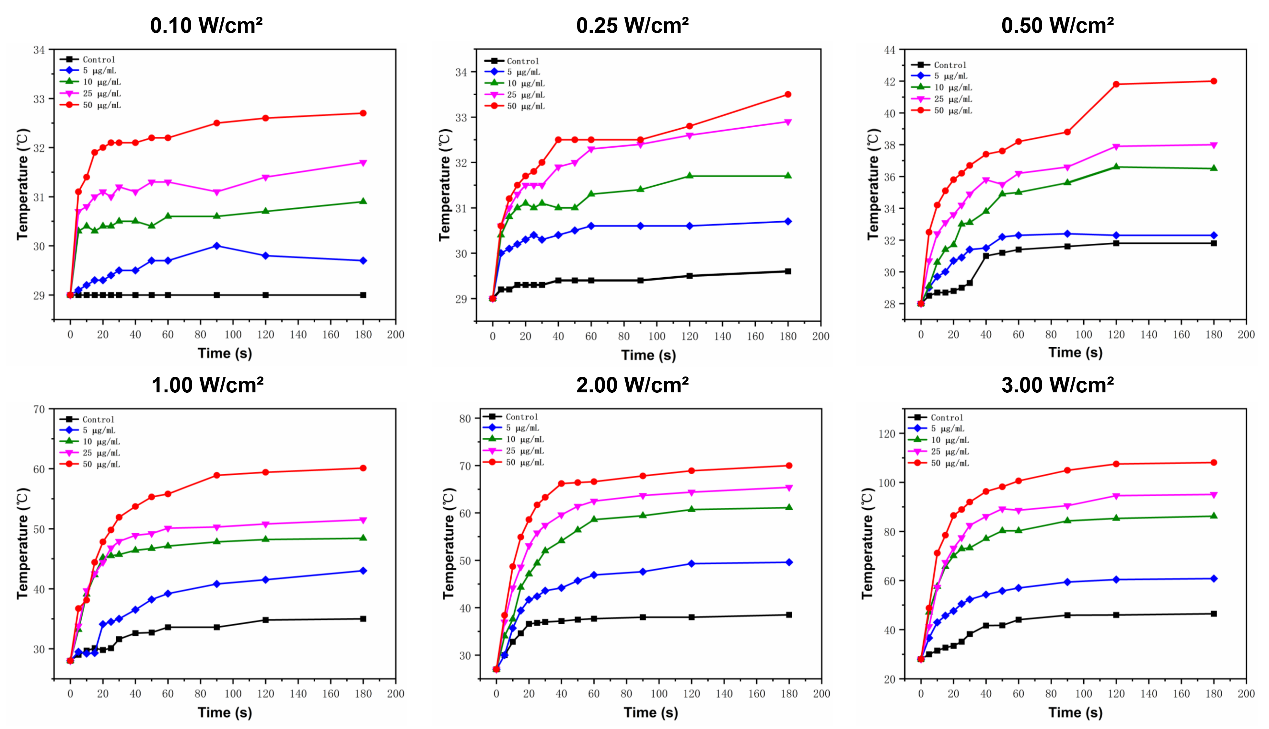


**Supplementary Figure 6**. Temperature curves of Rapa@Ti_3_C_2_-IOL with different concentrations of Ti_3_C_2_ under NIR irradiation. N = 3.


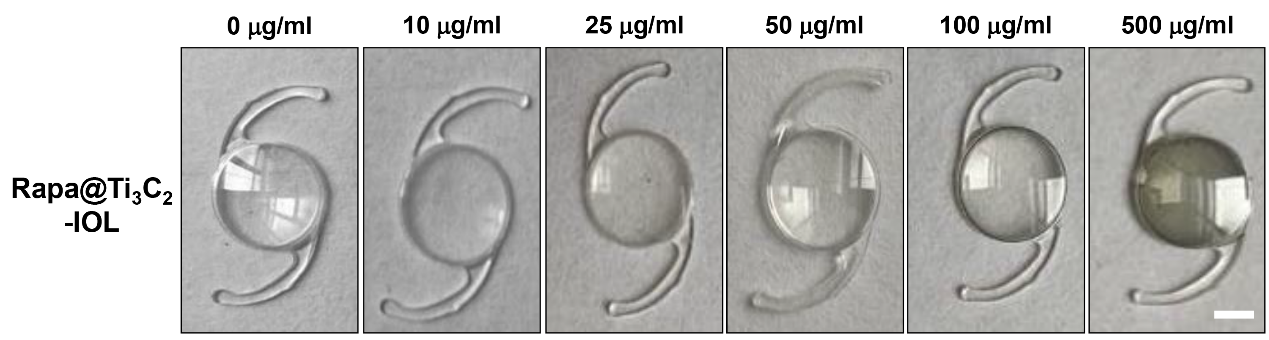


**Supplementary Figure 7**. The images of Rapa@Ti_3_C_2_-IOL with different concentrations of Ti_3_C_2_.


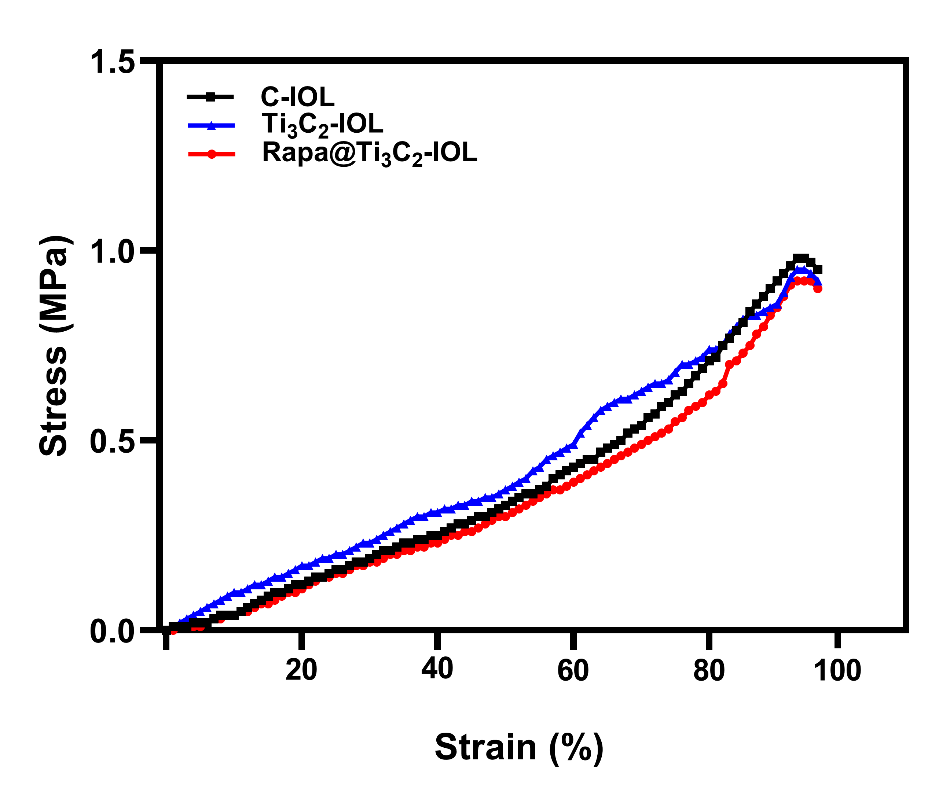


**Supplementary Figure 8**. The measurement of breaking elongation of C-IOL, Ti_3_C_2_-IOL and Rapa@Ti_3_C_2_-IOL.


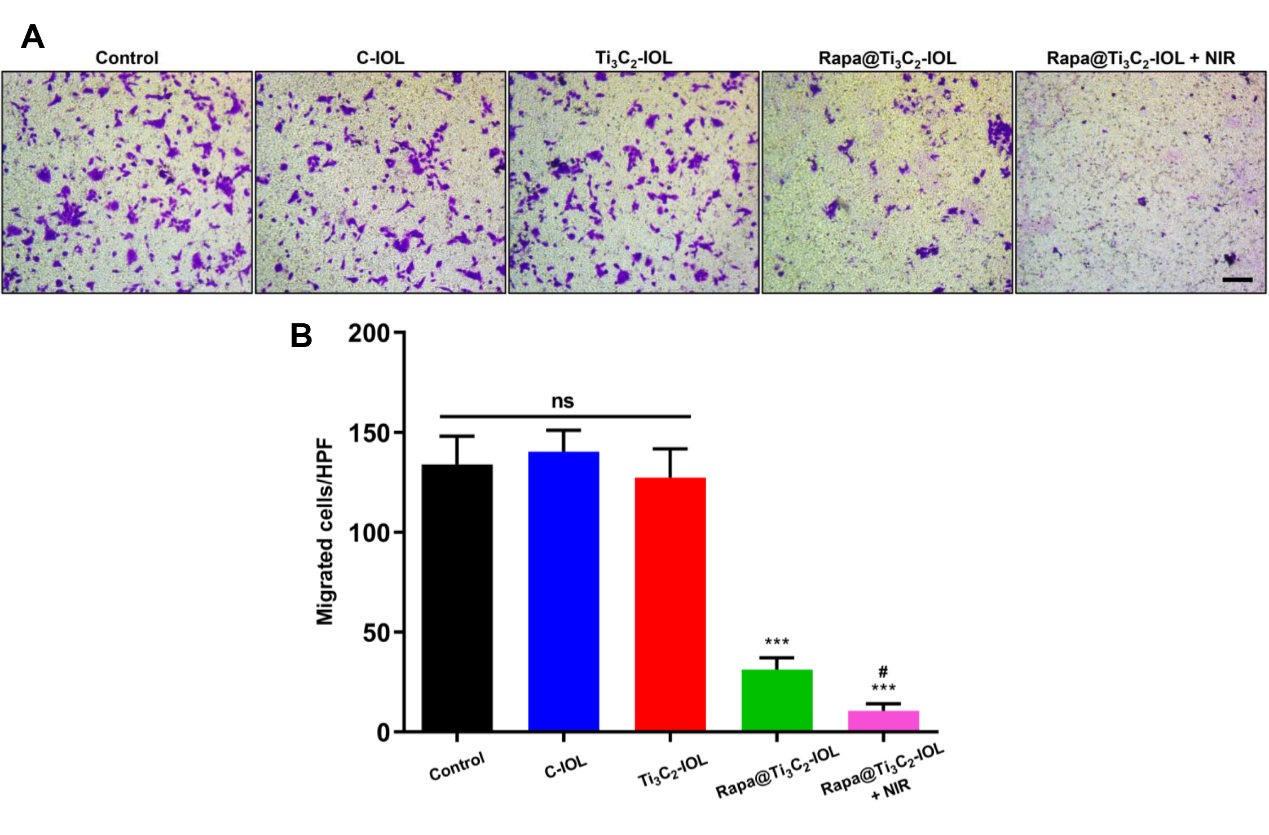


**Supplementary Figure 9**. The impact of different IOLs on cell migration ability of mouse LECs using Transwell migration assay. *** p<0.001 versus the control group. # p<0.05, versus Rapa@Ti_3_C_2_-IOL. N = 5. Scale bar = 50 µm.


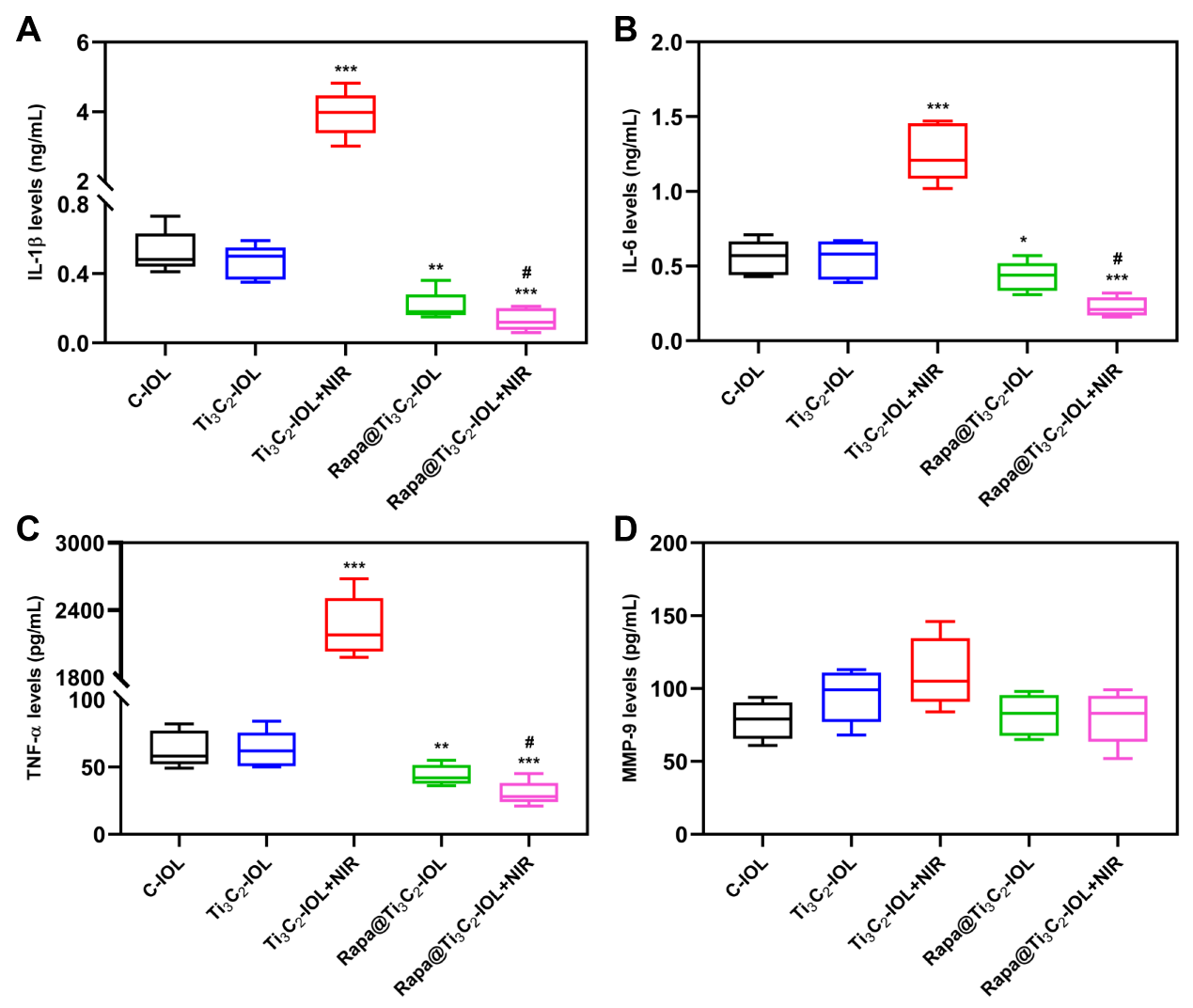


**Supplementary Figure 10**. The levels of IL-1β, IL-6, TNF-α and MMP-9 in the culture medium of each group.* p<0.05,** p<0.01,*** p<0.001 vs control IOL. # p<0.05, vs Rapa@Ti_3_C_2_-IOL. N = 4.


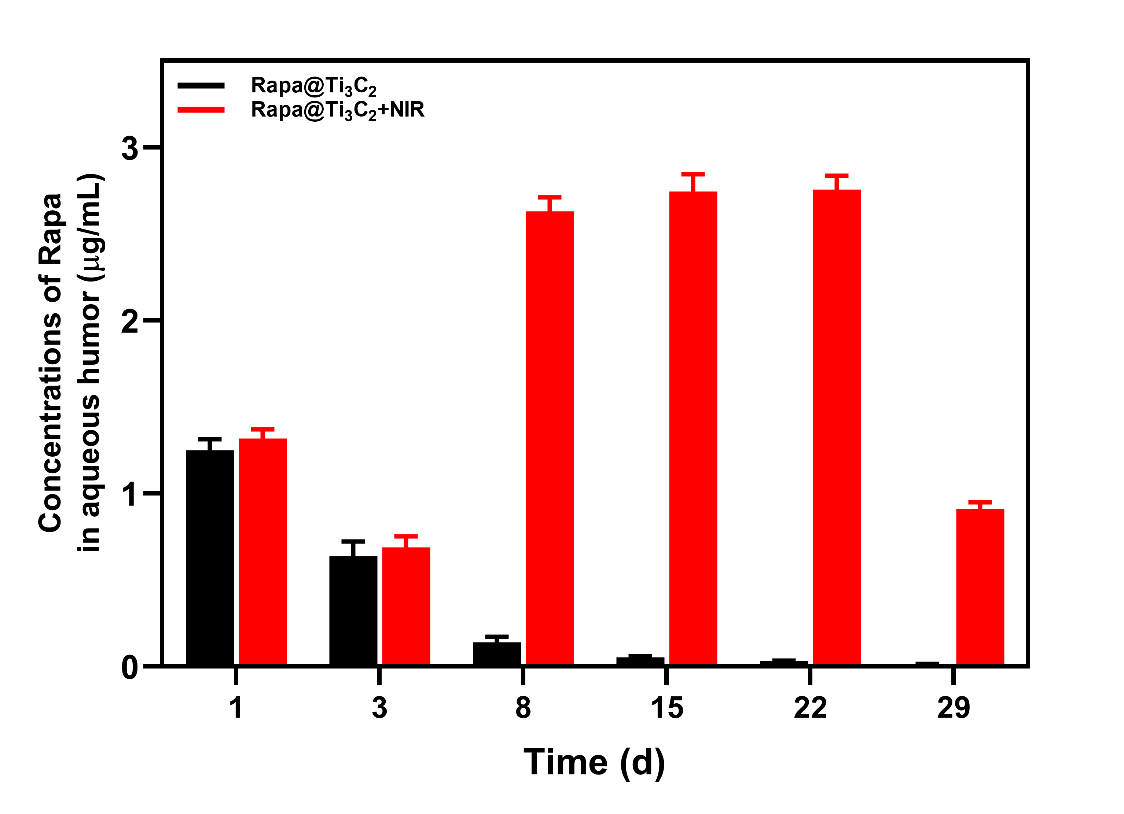


**Supplementary Figure 11**. Rapa concentrations in the aqueous humor of rabbits at the 1^st^, 3^rd^, 8^th^, 15^th^, 22^nd^, and 29^th^ day after Rapa@Ti_3_C_2_-IOL implantation. N = 5.


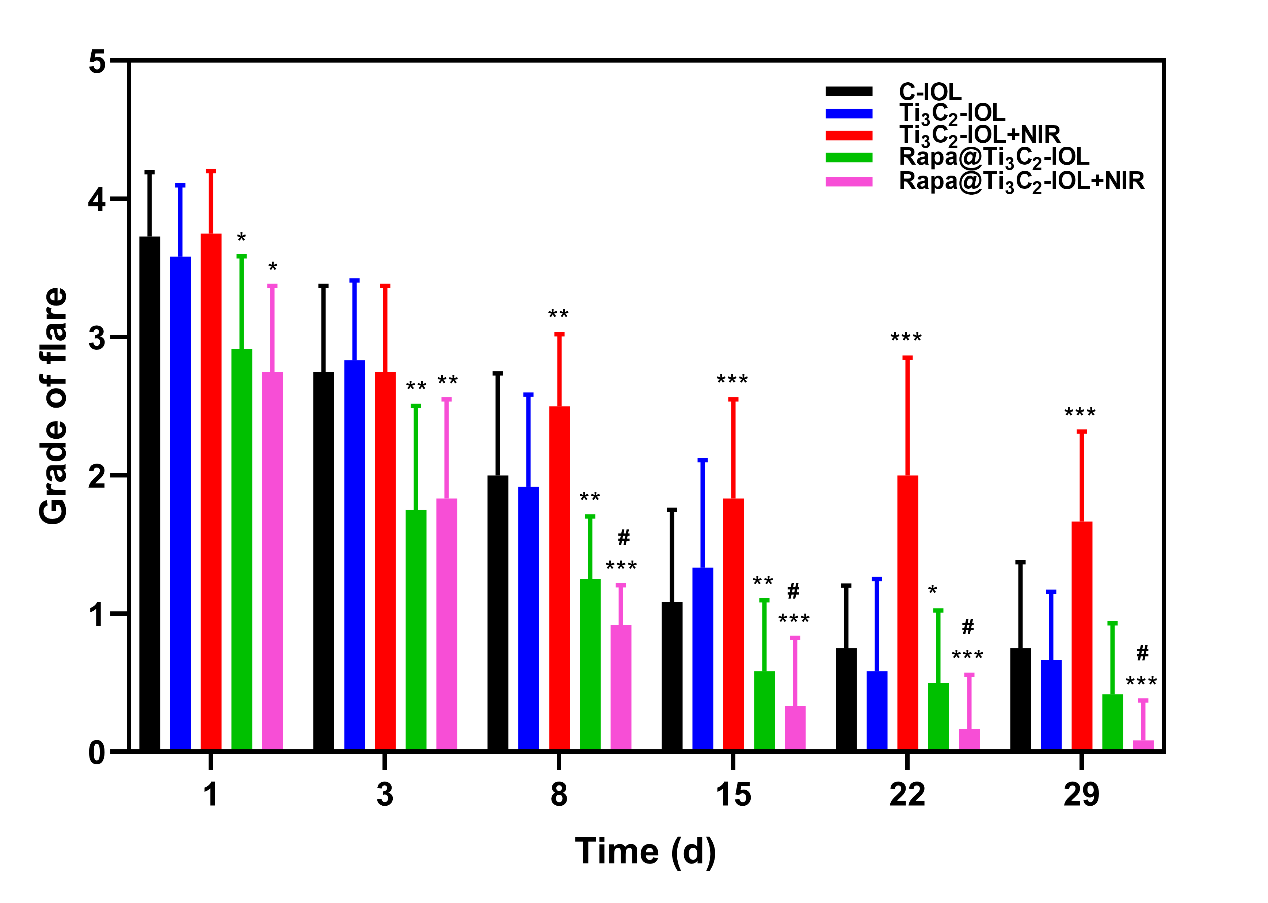


**Supplementary Figure 12**. Degree of anterior chamber flare on the 1^st^, 3^rd^, 8^th^, 15^th^, 22^nd^, and 29^th^ day after surgery. N = 12. *p<0.05, **p<0.01, ***p<0.001, versus C-IOL group. # p<0.05, versus Rapa@Ti_3_C_2_-IOL group.


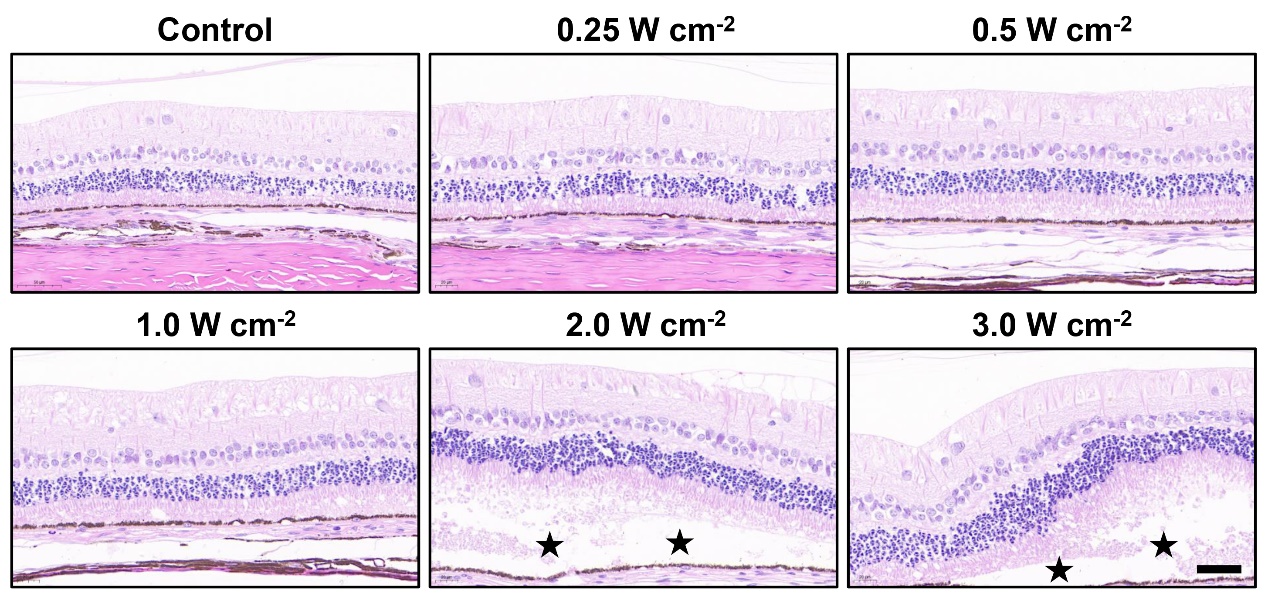


**Supplementary Figure 13**. H&E staining of the retina when the NIR power density ranges from 0 to 3.0 W/cm^2^. represents subretinal effusion and retinal detachment. N = 5. Scale bar = 100 µm.


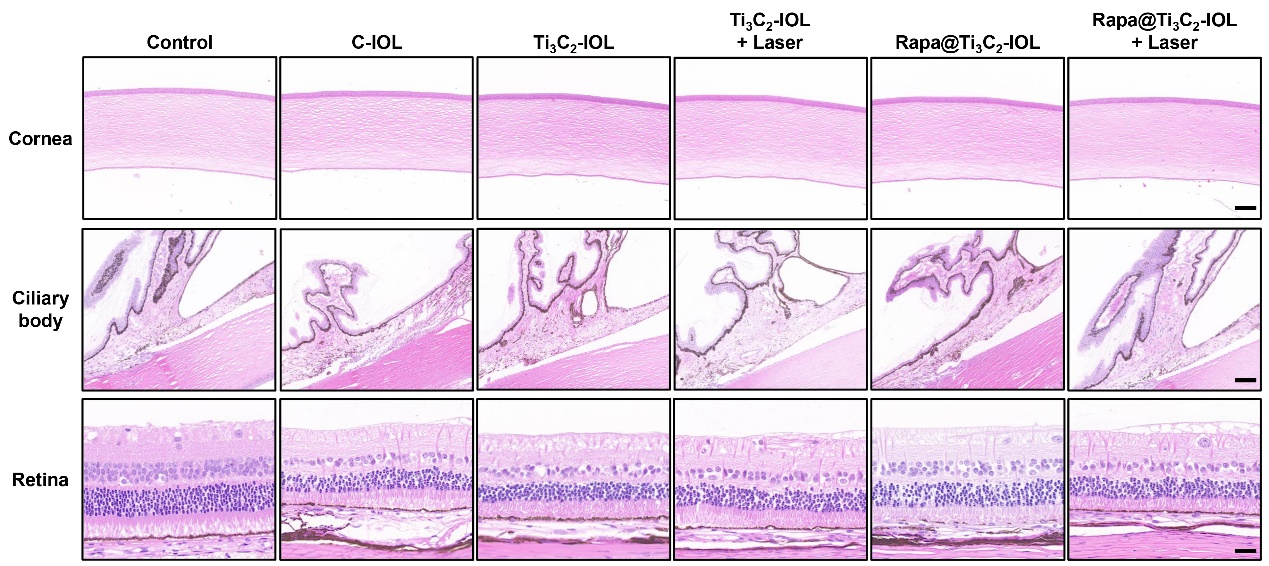


**Supplementary Figure 14**. H&E staining of the cornea, ciliary body and retina in different groups on day 28 after surgery. N = 5. Scale bar = 50 µm.


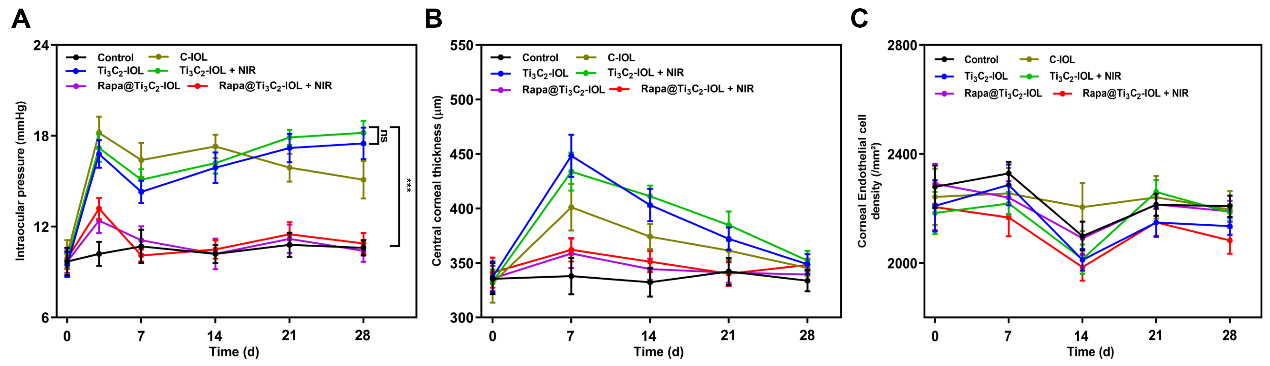


**Supplementary Figure 15**. Intraocular pressure, central corneal thickness and corneal endothelial cells of different IOL groups in each time point after surgery. *** p<0.001, versus the Rapa@Ti_3_C_2_-IOL+NIR group. N = 5.
